# Triple‐Negative Breast Cancer Cells Utilize IL8 and CXCL1 to Suppress NK Cells’ Function and Facilitate Cancer Metastasis

**DOI:** 10.1002/advs.75655

**Published:** 2026-05-25

**Authors:** Mingheng Yuan, Hongmei Yang, Renfei Wu, Meng Hao, Xiangpeng Chu, Chuxia Deng, Kathy Qian Luo

**Affiliations:** ^1^ Department of Biomedical Sciences Faculty of Health Sciences University of Macau Taipa Macao SAR China; ^2^ Ministry of Education Frontiers Science Center For Precision Oncology University of Macau Taipa Macao SAR China

**Keywords:** co‐culture, C‐X‐C motif chemokine ligand 1 (CXCL1), interleukin 8 (IL8), metastasis, natural killer (NK) cell, reactive oxygen species (ROS), triple‐negative breast cancer (TNBC)

## Abstract

Triple‐negative breast cancer (TNBC) is the most aggressive breast cancer subtype with high metastatic potential and limited treatment options. Natural killer (NK) cells represent a promising immunotherapy strategy due to their innate tumor‐killing capacity, but their efficacy against TNBC remains limited. We found that TNBC cells, particularly the mesenchymal‐like subtype, exhibited greater resistance to NK cells compared to non‐TNBC cells. Mechanistic studies indicate that TNBC cells’ survival in response to NK cells occurs in three phases. First, within 1 h of NK cell co‐culture, TNBC cells accumulate reactive oxygen species (ROS), which upregulate C‐X‐C motif chemokine ligand 1 (CXCL1) and interleukin 8 (IL8) expression in an NF‐κB− and ERK/JNK−AP‐1‐dependent manner. Second, secreted CXCL1/IL8 binds to C‐X‐C motif chemokine receptor 1/2 (CXCR1/2), activating the AKT−BCL‐2 pathway to enhance cancer cell survival and suppress NK cell function by downregulating NKG2D, TRAIL, and IFN‐γ expression. Third, CXCL1/IL8−CXCR1/2 autocrine loop further amplifies their own synthesis and induces programmed cell death 1 ligand 1 (PD‐L1) expression via NF‐κB and ERK/JNK−AP‐1 pathways. High CXCL1/IL8 expression correlates with reduced NK cell infiltration and shorter distant‐metastasis‐free survival in breast cancer patients. Combinatorial application of CXCR1/2 inhibitor with anti‐PD‐L1 antibody can overcome NK cell dysfunction and reduce TNBC metastasis.

## Graphical abstract

Triple‐negative breast cancer (TNBC) cells evade natural killer (NK) cell immunity by secreting IL8 and CXCL1. These chemokines suppress NK cells’ function via CXCR1/2 and enhance cancer cells’ survival through PD‐L1 upregulation and BCL‐2 anti‐apoptotic signaling. Combined inhibition of CXCR1/2 and PD‐L1 restores NK cell cytotoxicity and reduces metastasis, revealing a targetable immune‐evasion axis in TNBC.

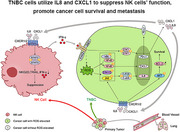

## 1 Introduction

According to the American Cancer Society, breast cancer remains the most commonly diagnosed cancer and the second leading cause of cancer‐related death among women in the United States in 2025 [1]. Triple‐negative breast cancer (TNBC) is the most aggressive breast cancer subtype, with a five‐year survival rate of 58% versus 72% for non‐TNBC patients [2, 3]. When metastasis develops within three months of diagnosis, the five‐year survival rate falls sharply to 25% [4]. Metastasis is driven by multiple processes, including epithelial‐mesenchymal transition (EMT), therapeutic resistance, and immune evasion, with the latter acts as a critical factor in tumor progression and dissemination [5, 6, 7].

During dissemination, cancer cells encounter immune surveillance from natural killer (NK) cells, macrophages, and T cells [8, 9]. Among these, NK cells are considered the first line of defense against tumor metastasis without prior sensitization or antigen presentation [10, 11]. Accumulating evidence indicates that TNBC cells can evade NK cell‐mediated cytotoxicity and achieve distant metastasis [12, 13]. Understanding the mechanisms by which TNBC cells evade NK cell‐mediated killing is critical for preventing early metastatic dissemination.

NK cell recognition depends on a dynamic balance between activating and inhibitory receptors [14, 15]. Inhibitory receptors, such as natural killer group 2, member A (NKG2A), engage major histocompatibility complex class I (MHC I) molecules to suppress NK activity. In contrast, activating receptors, such as natural killer group 2, member D (NKG2D), recognize stress‐induced ligands like MHC class I chain‐related molecules A and B (MICA/B), which transmit signals that trigger NK cell cytotoxicity [16, 17]. Based on current research, tumor evasion of NK cytotoxicity occurs through two major strategies. The first, termed “do not activate” strategy, enables tumor cells to evade NK cell recognition by concealing or modulating the expression of specific receptor ligands. For instance, breast cancer stem cells downregulate MICA/B expression, preventing recognition by NKG2D receptors on NK cells [18, 19, 20]. Another example is the expression of endogenous human leukocyte antigen G (HLA‐G) isoforms on glioma, melanoma, and renal carcinoma cells that deliver “do not kill me” signals to NK cells [21, 22, 23].

The second strategy involves remodeling the immunosuppressive tumor microenvironment (TME). For example, TNBC cells secrete chemokines such as C‐C motif chemokine ligand 2 (CCL2) to recruit regulatory T cells (Tregs), myeloid‐derived suppressor cells (MDSCs), and M2‐polarized macrophages [24, 25, 26, 27, 28]. These immunosuppressive cells release transforming growth factor beta (TGF‐β) and interleukin‐10 (IL‐10), further inhibiting NK cell activity [29, 30, 31]. Tumor cells also directly suppress NK cell cytotoxicity through the secretion of cytokines or metabolic byproducts [32, 33]. For instance, acute myeloid leukemia (AML) can induce excessive lactate accumulation in the TME, which promotes lysine lactylation in NK cells. This modification causes mitochondrial fragmentation and disrupted nicotinamide adenine dinucleotide (NAD+) metabolism, ultimately impairing NK cell cytotoxicity [34]. Cancer cells also secrete TGF‐β and interleukin‐6 (IL‐6) to inhibit granzyme B and perforin production in NK cells, thereby suppressing their cytotoxic function [30, 35, 36]. In addition, cancer cells can secrete glutamate‐leucine‐arginine (ELR)‑positive CXC chemokines to inhibit anti‐tumorigenic immune cells and promote cancer immune evasion [37]. However, the specific contribution of individual ligands to cancer immune evasion, particularly in TNBC, remains poorly defined.

Despite these advances, most research has focused on how tumor cells generally suppress NK cells or how to enhance NK cell cytotoxicity, with limited focus on how TNBC cells actively reprogram their survival and metastatic potential after direct interaction with NK cells. Crucially, the specific mechanisms by which TNBC cells dynamically adapt to NK cell pressure, particularly gaining survival and metastatic traits, remain largely unexplored. To address this, we co‐cultured various breast cancer cell lines with NK cells and observed that human epidermal growth factor receptor 2 (HER2)‐positive and TNBC cells, especially the mesenchymal‐like subtypes of TNBC cells, survived better than estrogen receptor (ER)‐positive cells. Transcriptomic profiling identified TNBC‐specific driver genes that impair NK cell cytotoxicity and enhance tumor cell survival. With these insights, we investigated how NK cells induce these genes and how they influence both cancer cells and NK cells. Our study highlights a therapeutic strategy to prevent TNBC metastasis and improve immunotherapy efficacy.

## 2 Results

### 2.1 High Metastatic Breast Cancer (HMBC) Cells Survived Better Than Low Metastatic Breast Cancer (LMBC) Cells During Co‐Culture With NK Cells

Breast cancer cells are generally categorized as ER‐positive, HER2‐positive, and TNBC. Among these, TNBC and HER2‐positive subtypes exhibit more aggressive growth and a greater propensity for metastasis, which may be collectively referred to as high metastatic potential breast cancer (HMBC). In contrast, ER‐positive tumors show lower metastatic potential and are defined as low metastatic potential breast cancer (LMBC). To compare their differential cytotoxic sensitivity to NK cells, we co‐cultured HMBC and LMBC cell lines with NK‐tdT cells in ultralow‐adhesion round‐bottom 96‐well plates for 48 h. The TNBC group included two mesenchymal‐like (ML) subtypes, specifically MDA‐MB‐231‐GFP (231‐GFP) and BT549‐clover, as well as two basal‐like 1 (BL1) subtypes, namely MDA‐MB‐468‐clover (468‐clover) and HCC1937‐clover [38]. HER2^+^ breast cancer cells (BT474‐clover) and ER^+^/HER2^−^ breast cancer cells (MCF7‐C3 and T47D‐clover) were also tested for NK cell resistance. All breast cancer cells were transfected with fluorescent reporters (GFP, clover, and caspase‐sensor C3) for viability tracking as previously described [39]. Cancer cell viability was measured by green fluorescence, whereas NK cell viability was tracked by the tandem dimer Tomato (tdT) red fluorescent protein signal.

**FIGURE 1:**
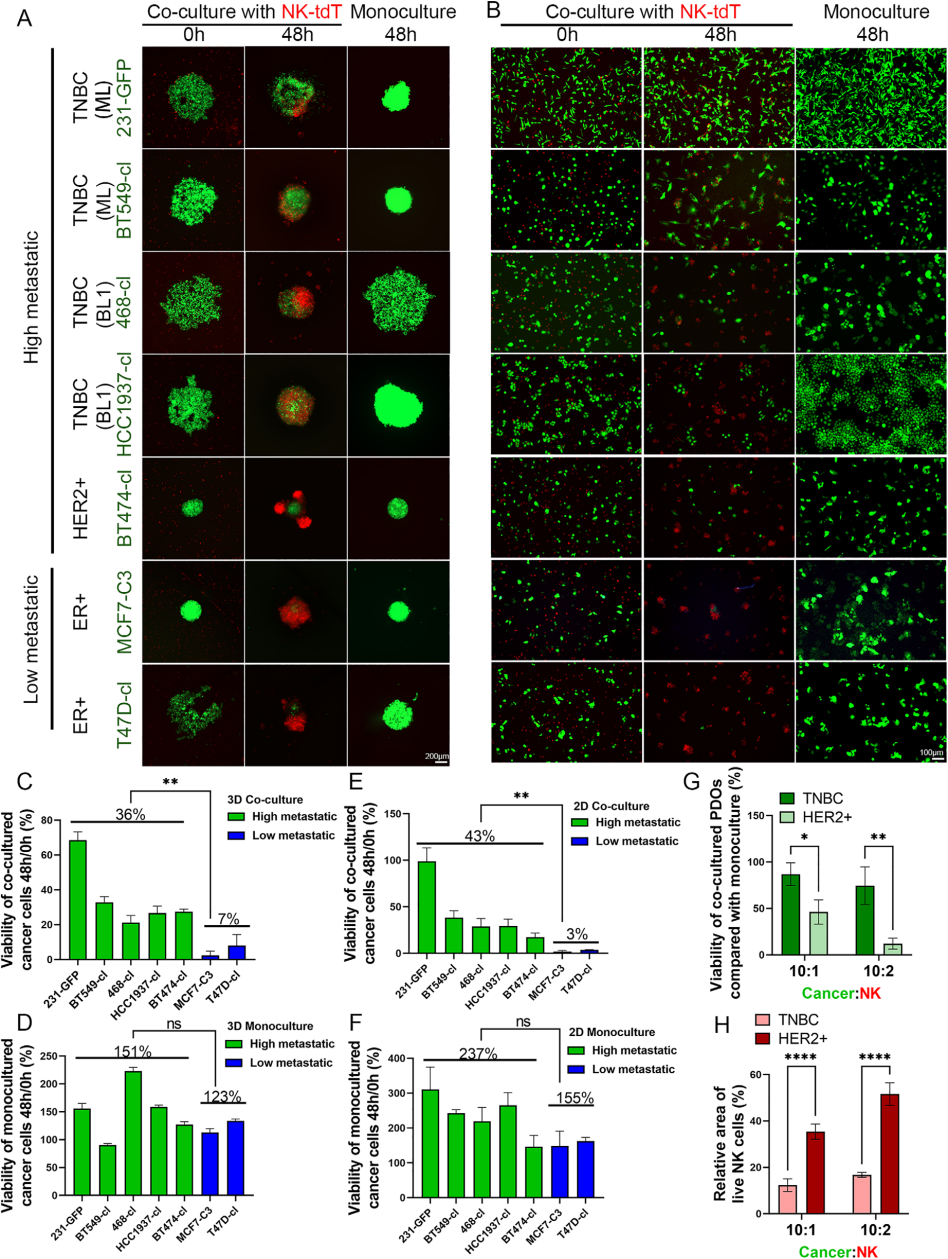
HMBC cells resist NK cell‐mediated killing more effectively than LMBC cells. (A) Representative 3D co‐culture images of breast cancer subtypes with NK cells (cancer‐to‐NK ratio of 2:1 for 48 h). Scale bar, 200 µm. (B) Representative 2D co‐culture images of breast cancer subtypes with NK cells (cancer‐to‐NK ratio of 2:1 for 48 h). Scale bar, 100 µm. (C–F) Quantification of cancer cell survival under co‐culture or monoculture conditions. (G, H) Quantitative analysis of the viability of patient‐derived organoids (PDOs) from HER2‐positive and TNBC patients co‐cultured with NK‐tdT cells at cancer‐to‐NK ratios of 10:1 or 10:2 for 72 h. Viability of cancer cells was assessed using Calcein‐AM staining (G). The percentage of live NK cell area was determined by measuring red fluorescence area (H). The results represent the means ± SD from three independent experiments. Significant differences were determined by Student's *t*‐test or two‐way ANOVA. *p < 0.05, **p < 0.01, ****p < 0.0001, ns, not significant.

In 3D co‐culture, HMBC cells exhibited superior survival to LMBC cells after 48 h (Figure 1A). The average viability of HMBC cells was 36% compared to 7% for LMBC cells, representing a greater than five‐fold elevation relative to that of LMBC cells (Figure 1C). Similar results were obtained in a 2D co‐culture condition (Figure 1B). After 48 h of 2D co‐culture, the average viability of HMBC cells was 43% compared to 3% of LMBC cells, over 14‐fold higher viability (Figure 1E). Among the four TNBC cell lines, 231‐GFP showed approximately two‐fold stronger cell viability than the other three TNBC cell lines in both 3D and 2D co‐cultures. In monoculture, no difference was found between HMBC and LMBC cells in both 3D and 2D conditions, suggesting that NK cell resistance of HMBC cells was not due to growth rate (Figure 1D,F). We also employed the calcein‐AM assay to evaluate the NK cell cytotoxicity against breast cancer cell lines. Compared to LMBC cells, HMBC cells exhibited greater resistance to NK cell‐mediated lysis at multiple ratios between cancer and NK cells, including 1:2, 1:1, 2:1, and 5:1 (Figure S1A). Among these four ratios, at a 1:1 ratio for 6 h, NK cells lysed 29% of LMBC cells versus 7% of HMBC cells, which was more than a four‑fold stronger killing effect against LMBC cells (Figure S1B).

Next, we used patient‐derived organoids to investigate the killing effects of NK cells on human TNBC and HER2^+^ breast cancer tissues. TNBC‐derived organoids co‐cultured with NK cells at the ratio of 10:2 exhibited 6‐fold higher viability than HER2‐positive organoids (Figure 1G and Figure S1C,D). Importantly, the number of NK cells in the TNBC‐derived PDOs measured by their red fluorescence intensity was approximately three‐fold lower than the numbers in the HER2^+^ PDOs after 72 h of co‐culture (Figure 1H and Figure S1C,D). This phenotype mirrors our observations in established cell lines, demonstrating that NK cell resistance is also present in tumor cells derived from primary tumors of TNBC patients, and cancer cells derived from TNBC organoids can also impair the viability of NK cells.

Interestingly, after 48 h of 3D co‐culture, NK cell proliferation was 1.5‐fold higher with non‐TNBC cells than with TNBC cells. Specifically, co‐culturing with ML‐TNBC showed a stronger inhibitory effect on NK activity than co‐culture with BL1‐TNBC (Figure S2F). This suggests TNBC survival is aided by suppressing NK cell proliferation, especially for ML‐TNBC cells. We hypothesized that TNBC cells secrete cytokines to suppress NK cell function. To test this hypothesis, we treated NK cells with conditioned medium (CM) of various breast cancer cell lines. The viability of NK cells treated with TNBC‐derived CM was 1.5‐fold lower than that of non‐TNBC CM (Figure S2A,B). The results of the EdU incorporation assay further confirmed that the proliferation of NK cells was 1.4‐ to 2.2‐fold lower when cultured in the conditioned medium of ML‐TNBC (231‐GFP and BT549‐clover) cells compared with cultured in the conditioned medium of non‐TNBC MCF‐7 cells (Figure S2C,D). In addition, NK cells secreted less interferon‐γ (IFN‐γ) when co‐cultured with 231‐GFP than MCF7‐C3 cells, indicating that 231‐GFP cells produced a stronger inhibitory effect on the cytotoxic function of NK cells (Figure S2E).

### 2.2 IL8 and CXCL1 Were Enriched in TNBC Cells and Negatively Correlated With NK Cell Infiltration in Human Samples

**FIGURE 2:**
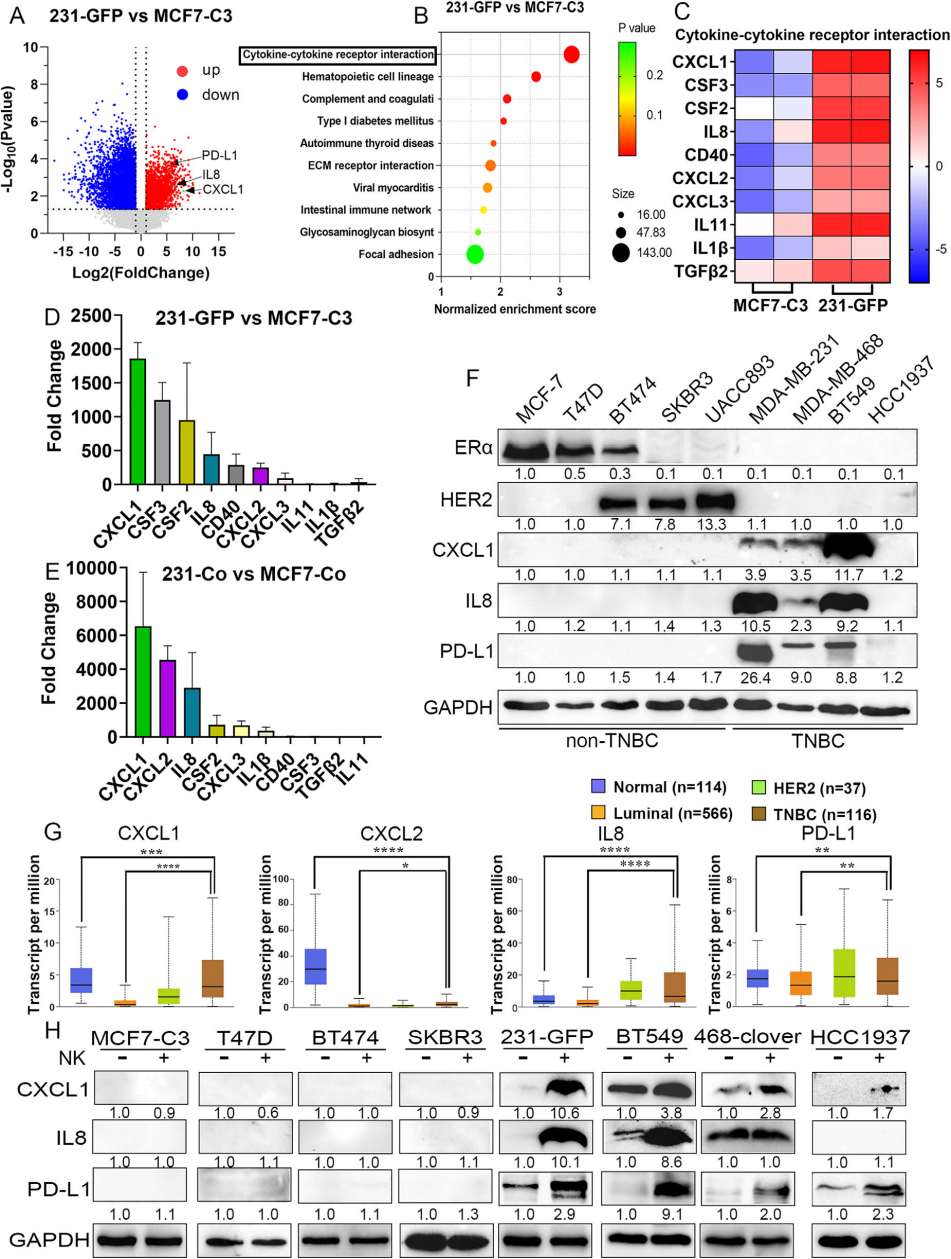
IL8, CXCL1, and PD‐L1 were enriched in TNBC cells and further upregulated upon co‐culture with NK cells. (A) Volcano plot showing the differentially expressed genes between 231‐GFP and MCF7‐C3 cells. (B) GSEA showing enrichment of cytokine‐cytokine receptor pathway in 231‐GFP cells. (C) Heatmap displaying the top 10 differentially expressed cytokines. (D, E) qPCR validation of 10 cytokine genes in 231‐GFP vs MCF7‐C3 cells before (D) or after (E) co‐culture with NK cells. (F) Western blot results showing the expression levels of CXCL1, IL8, and PD‐L1 in nine breast cancer cell lines. (G) Expression levels of CXCL1, CXCL2, IL8, and PD‐L1 in human normal and breast cancer tissues (BRCA) based on breast cancer subclasses from TCGA samples, *n* = 833 samples (UALCAN webtool). H) Protein levels in breast cancer cell lines before and after co‐culture with NK cells. The results represent the means ± SD from three independent experiments. Significant differences were identified using Student's t‐test. *p < 0.05, **p < 0.01, ***p < 0.001, ****p < 0.0001.

To identify driver genes for enhancing NK cell resistance in 231‐GFP versus MCF7‐C3 cells, we performed RNA sequencing on both cell lines. Transcriptome analysis revealed 2105 genes significantly upregulated in 231‐GFP cells (Figure 2A). Next, Gene Set Enrichment Analysis (GSEA) highlighted the “cytokine‐cytokine receptor interaction” as the most enriched pathway in 231‐GFP cells (Figure 2B). From this pathway, we selected the top 10 most significant cytokines (Figure 2C) for validation using quantitative polymerase chain reaction (qPCR). Seven out of ten genes were significantly upregulated in 231‐GFP compared to MCF7‐C3 cells (Figure 2D). After co‐culture with NK cells, CXCL1, CXCL2, and IL8 displayed the highest levels of upregulation in 231‐GFP cells compared to MCF7‐C3 cells, suggesting their potential roles in helping 231‐GFP cells resist the cytotoxicity of NK cells (Figure 2E).

We further measured the mRNA levels of the 10 selected genes among nine cancer cell lines representing different types of breast cancer by qPCR. The gene expression levels of CXCL1, CXCL2, and IL8 were highly enriched in TNBC cell lines of MDA‐MB‐231, MDA‐MB‐468, and BT549, with average relative mRNA levels in these three TNBC cells exceeding a 980‐fold increase relative to MCF‐7 cells (Figure S3A). Based on prior evidence that PD‐L1 aids immune evasion from NK cells [40, 41, 42], we also measured its expression level and found that it was also enriched in TNBC cell lines of MDA‐MB‐231 and BT549 (Figure S3A). We then determined the protein levels of these 11 proteins among four TNBC cell lines and five non‐TNBC cell lines using the data from The Human Protein Atlas database. The results showed that the expression levels of CXCL1, IL8, and PD‐L1 were much higher in the TNBC cell lines than in the non‐TNBC cell lines (Figure S3B).

Western blot results further confirmed that the protein levels of CXCL1, IL8, and PD‐L1 were much higher in cell lines of ML‐TNBC subtype (MDA‐MB‐231 and BT549) than in cell lines of BL1‐TNBC subtype (MDA‐MB‐468 and HCC1937), consistent with their stronger NK‐resistant phenotype (Figure 2F). By analysing the data from UALCAN database, we found that the expression of CXCL1, IL8, and PD‐L1 was significantly higher in patients with TNBC than in patients with luminal breast cancer or normal breast tissues (Figure 2G).

Further analysis of the RNA‐seq data revealed that the transcript levels of CXCL1 (GROα) and IL8 (CXCL8) are at the highest fragment per kilobase million (FPKM) (74.14 and 72.82), while other members of CXCL family including CXCL2 and CXCL3 displayed much lower transcript levels, and CXCL5, CXCL6 and CXCL7 showed minimum levels of expression in 231‐GFP cells compared to that in MCF7‐C3 cells (Figure S4A). We also found that transcriptional expressions of CXCL2 and CXCL3 are even higher in normal breast tissues compared to the levels in TNBC tumors from clinical samples (Figure 2G and Figure S4B), we therefore decided to select CXCL1, IL8, and PD‐L1 as the candidate genes for further investigation in this study.

We then compared the protein levels of these three candidate genes in eight different cell lines before and after they were co‐cultured with NK cells by Western blot analysis. TNBC cells exhibited markedly higher upregulation than non‐TNBC cells, with average increases of ∼5‐fold in CXCL1 and IL8, and approximately four‐fold in PD‐L1. Specifically, ML‐TNBC cells (231‐GFP and BT549) showed even greater upregulation than BL1‐TNBC cells (468‐clover and HCC1937). On average, 231‐GFP and BT549 showed approximately nine‐fold upregulation of IL8 and three‐fold upregulation of CXCL1 and PD‐L1 compared with 468‐clover and HCC1937 (Figure 2H). In human breast cancer tissues, the mRNA levels of CXCL1, IL8, and PD‐L1 genes were positively correlated with each other and negatively correlated with NK cell infiltration, suggesting a cooperative suppression of NK cell function (Figure S4C–E). In summary, we identified IL8, CXCL1, and PD‐L1 as three potential driver genes associated with TNBC resistance to NK cell cytotoxicity.

### 2.3 Knockdown of IL8 and CXCL1 Reduced PD‐L1 Expression and Increased NK Cell Cytotoxicity Against TNBC Cells

**FIGURE 3:**
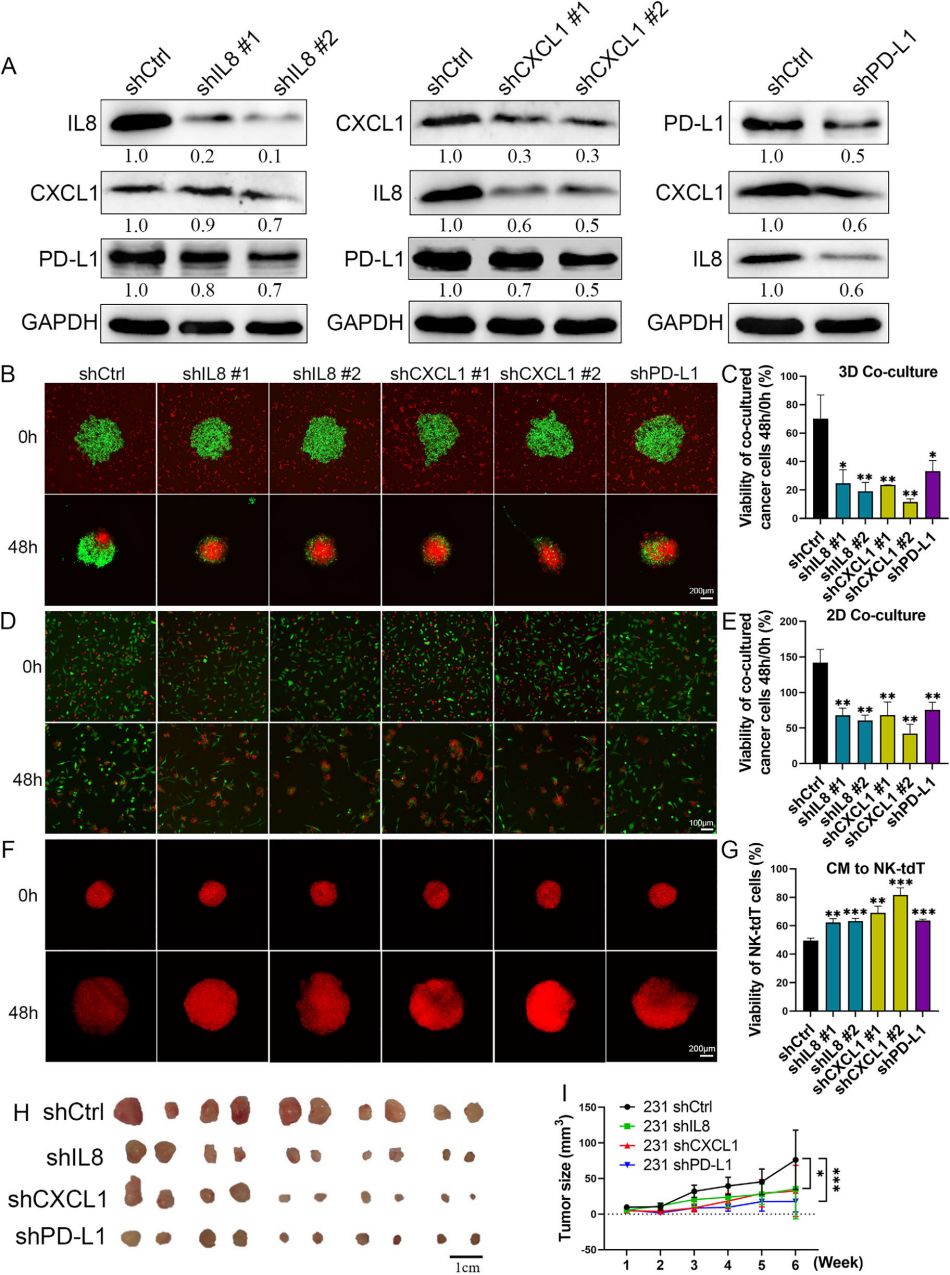
Knockdown of IL8, CXCL1, or PD‐L1 sensitized 231‐GFP cells to NK cell killing. (A) Western blot analysis showing knockdown of a single gene reduced levels of the others. (B, C) Reduced survival of TNBC cells after knockdown in 3D co‐culture with NK cells at a ratio of 2:1 for 48 h. Scale bar, 200 µm. (D, E) Reduced survival of TNBC cells after knockdown in 2D co‐culture with NK cells at a ratio of 2:1 for 48 h. Scale bar, 100 µm. (F, G) Conditioned medium (CM) from knockdown cells enhanced NK cell proliferation. Scale bar, 200 µm. (H, I) Representative images and tumor size measurements of orthotopic tumors formed by 231‐GFP cells with IL8, CXCL1, and PD‐L1 knockdown in nude mice after six weeks (*n* = 5 mice per group). Scale bar, 1 cm. The results represent the means ± SD from three independent experiments. Significant differences were determined by one‐way or two‐way ANOVA. *p < 0.05, **p < 0.01, ***p < 0.001.

To assess the functional roles of IL8, CXCL1, and PD‐L1 in facilitating NK cell resistance, we knocked down each gene in 231‐GFP and BT549‐clover cells using shRNA. IL8, CXCL1, and PD‐L1 mutually positively regulate each other's expression, as the knockdown of any one gene reduced the expression of the other two (Figure 3A). Consistent effects were observed in BT549‐clover cells with IL8, CXCL1, or PD‐L1 knockdown (Figure S5A–C).

Next, we co‐cultured NK‐tdT cells with these knockdown cells. In 3D co‐culture, the viability of 231‐GFP cells decreased by 73% (shIL8 #2), 84% (shCXCL1 #2), and 53% (shPD‐L1) (Figure 3B,C). Similarly, BT549‐clover knockdown also reduced cell viability by 66% (shIL8 #2), 81% (shCXCL1 #2), and 75% (shPD‐L1) (Figure S5D,E). Comparable trends were observed in 2D co‐culture, with 231‐GFP viability dropping by 57% (shIL8 #2), 70% (shCXCL1 #2), and 47% (shPD‐L1) (Figure 3D,E), and BT549‐clover by 38% (shIL8 #2), 57% (shCXCL1 #2), and 38% (shPD‐L1) (Figure S5F,G).

To investigate whether knockdown could reverse NK cell suppression by TNBC conditioned medium (CM), we measured NK cell viability in CM from TNBC knockdown cells. After 48 h of culturing, NK cell viability increased to ∼1.3‐fold (shIL8 #2), ∼1.7‐fold (shCXCL1 #2), and ∼1.3‐fold (shPD‐L1) in 231‐GFP CM, and to ∼4.4‐fold (shIL8 #2), ∼4.7‐fold (shCXCL1 #2), and ∼1.9‐fold (shPD‐L1) in BT549‐clover CM (Figure 3F,G and Figure S5H,I). These results confirm that CXCL1, IL8, and PD‐L1 contribute to TNBC resistance against NK cells and inhibit NK cell activity in vitro.

To investigate the role of these genes in cancer metastasis, we orthotopically injected knockdown cells into the mammary fat pads of nude mice, which lack T and B cells, while retaining functional and elevated NK cell activity. Knockdown of these three genes greatly reduced tumor size by 53% (shIL8), 57% (shCXCL1), and 77% (shPD‐L1) (Figure 3H,I), and tumor weight by 59% (shIL8), 60% (shCXCL1), and 79% (shPD‐L1) compared to shCtrl (Figure S5O). While no significant change in body weight was observed across 4 groups of mice (Figure S5P, Supporting Information). Importantly, iliac lymph node metastasis frequency decreased by 30–40%, and left lung metastatic colonies were reduced by 30%–50% (Figure S5J–N). In summary, IL8, CXCL1, and PD‐L1 exhibit positively correlated expression and collectively promote TNBC survival under NK cell pressure and facilitate metastasis in vivo.

### 2.4 Overexpression of IL8 and CXCL1 Enhanced PD‐L1 Expression and Attenuated NK Cell‐Mediated Cytotoxicity

**FIGURE 4:**
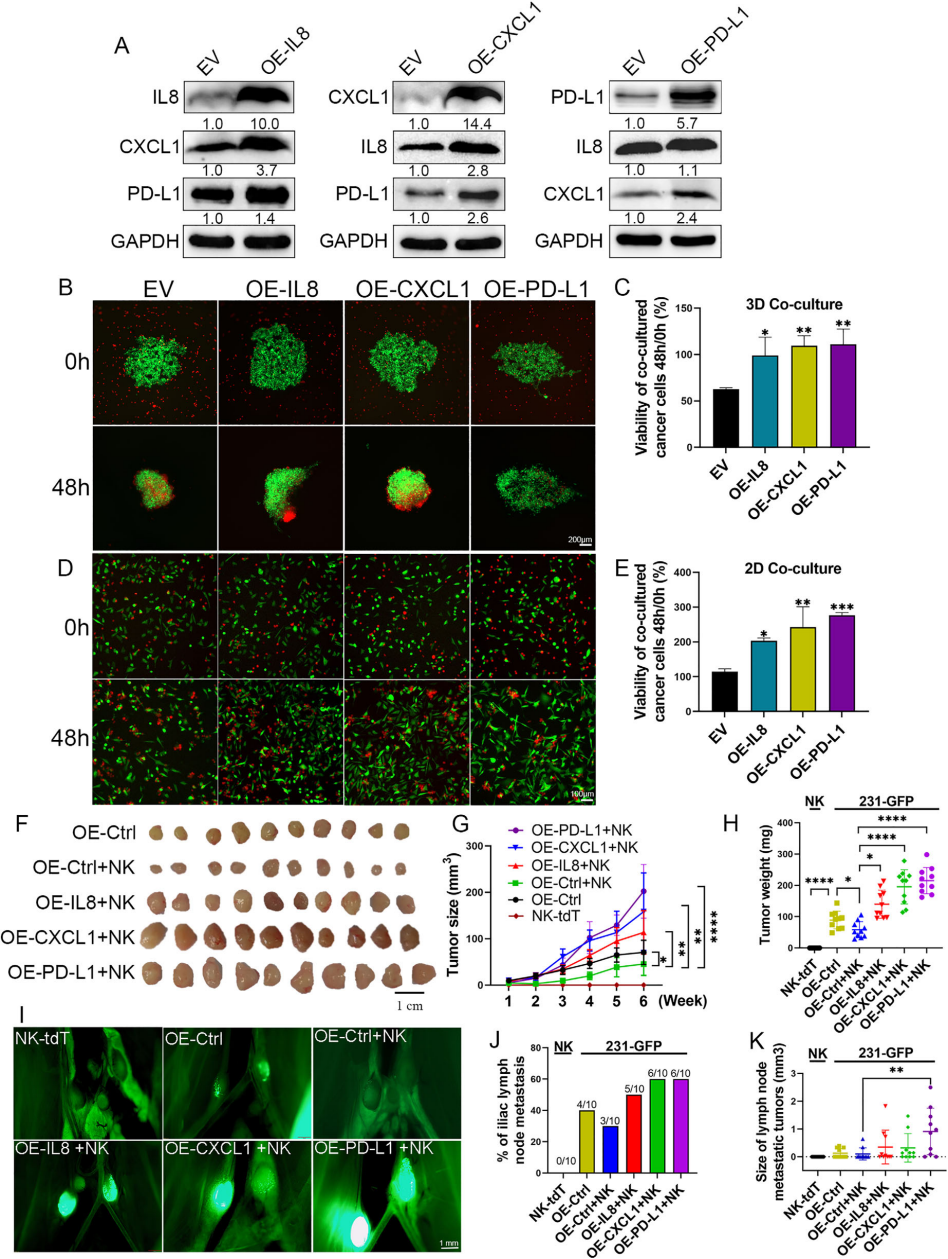
Overexpression of IL8, CXCL1, or PD‐L1 promoted 231‐GFP cell survival and metastasis. (A) Western blot analysis showing the protein levels of IL8, CXCL1, and PD‐L1 in overexpression cells. (B, C) Representative images and quantification of 3D co‐culture of overexpression 231‐GFP cells with NK cells at a ratio of 2:1 for 48 h. Scale bar, 200 µm. (D, E) Representative images and quantification of 2D co‐culture of overexpression 231‐GFP cells with NK cells at a ratio of 2:1 for 48 h. Scale bar, 100 µm. (F, G) Representative images and size measurements of orthotopic tumors formed by monocultured or co‐cultured 231‐GFP cells in NOD/SCID mice after six weeks (*n* = 5 mice per group). Scale bar, 1 cm. H) Tumor weights of breast primary tumors were measured on day 42. (I–K) Representative images and quantification of iliac lymph node metastasis. Scale bar, 1 mm. The results represent the means ± SD from three independent experiments. Significant differences were determined by Student's *t*‐test, one‐way or two‐way ANOVA. *p < 0.05, **p < 0.01, ***p < 0.001, ****p < 0.0001.

To further validate the roles of IL8, CXCL1, and PD‐L1 in protecting cancer cells against NK cells during the co‐culture, we overexpressed these genes in 231‐GFP, MCF7‐C3, and HCC1937‐clover cell lines (Figure 4A and Figure S6A,B,G). The Western blot results showed that overexpression of IL8 or CXCL1 increased the protein levels of all three genes, but overexpression of PD‐L1 only increased the protein level of CXCL1, but not that of IL8 (Figure 4A). Unlike the phenomena observed in 231‐GFP cells, overexpression of IL8 or CXCL1 in MCF7‐C3 cells only elevated their own gene expression, but did not affect the mRNA levels of the other two genes (Figure S6A,B).

Next, we assessed the viability of overexpressing cells following co‑culture with NK cells. Overexpression of IL8, CXCL1, and PD‐L1 increased the viability of 231‐GFP cells to 1.6‐fold (OE‐IL8), 1.7‐fold (OE‐CXCL1), and 1.8‐fold (OE‐PD‐L1) (Figure 4B,C) A bigger enhancement was observed in MCF7‑C3 cells, with increases of 2.8‑fold (OE‑IL8) and 3.4‑fold (OE‑CXCL1), and in HCC1937‑clover cells by approximately 2.5‑fold (Figure S6C,D,H,I). In 2D co‐culture conditions, overexpression of IL8, CXCL1, and PD‐L1 increased viability of 231‐GFP to 1.8‐fold (OE‐IL8), 2.1‐fold (OE‐CXCL1), and 2.4‐fold (OE‐PD‐L1) (Figure 4D,E), as well as MCF7‐C3 cells to 1.6‐fold (OE‐IL8) and 1.7‐fold (OE‐CXCL1) (Figure S6C,E).

Interestingly, overexpression of IL8 increased the inhibitory effect of conditioned medium (CM) from 231‐GFP and MCF7‐C3 cells on NK cells by 15% and 10%, respectively. Similarly, overexpression of CXCL1 enhanced NK cell suppression by 12% and 30%, indicating the inhibitory effect of IL8 and CXCL1. We noticed that overexpression of PD‐L1 did not significantly affect the growth of NK cells in the CM of 231‐GFP cells (Figure S6C,F,J,K).

To determine whether IL8, CXCL1, and PD‐L1 could enhance tumor resistance to NK cell cytotoxicity and promote distant metastasis, we co‐cultured control and overexpression 231‐GFP cells with NK‐tdT cells (5:1 ratio) for 24 h. The mixture of pre‐cultured 231‐GFP cells with NK‐tdT cells (5:1 ratio) was then injected into the mammary glands of NOD/SCID mice, which lack functional T cells, B cells, and NK cells, to establish an orthotopic model unaffected by host‐derived NK cell activity. At the end of 6‐weeks animal experiments, coculturing with NK cells decreased the tumor volume of OE‐Ctrl+NK cells by 35% compared with OE‐Ctrl cells. Overexpression of IL8, CXCL1, and PD‐L1 overcame NK‑mediated tumor suppression and increased tumor volume by 141%, 236%, and 270%, respectively (Figure 4F–H). Moreover, overexpression of these three genes elevated the incidence of iliac lymph node metastasis by 1.7‐ to 2‐fold and expanded the metastatic areas by 2.5‐fold (OE‐IL8), 2.2‐fold (OE‐CXCL1), and 8.1‐fold (OE‐PD‐L1) (Figure 4I–K). These results indicate that IL8, CXCL1, and PD‐L1 attenuate NK cell cytotoxicity and promote lymph node metastasis in TNBC cells.

### 2.5 Metastatic TNBC Cells Use the IL8/CXCL1−CXCR1/2 Pathway to Resist NK Cell‐Mediated Killing and Enhance Metastasis

**FIGURE 5:**
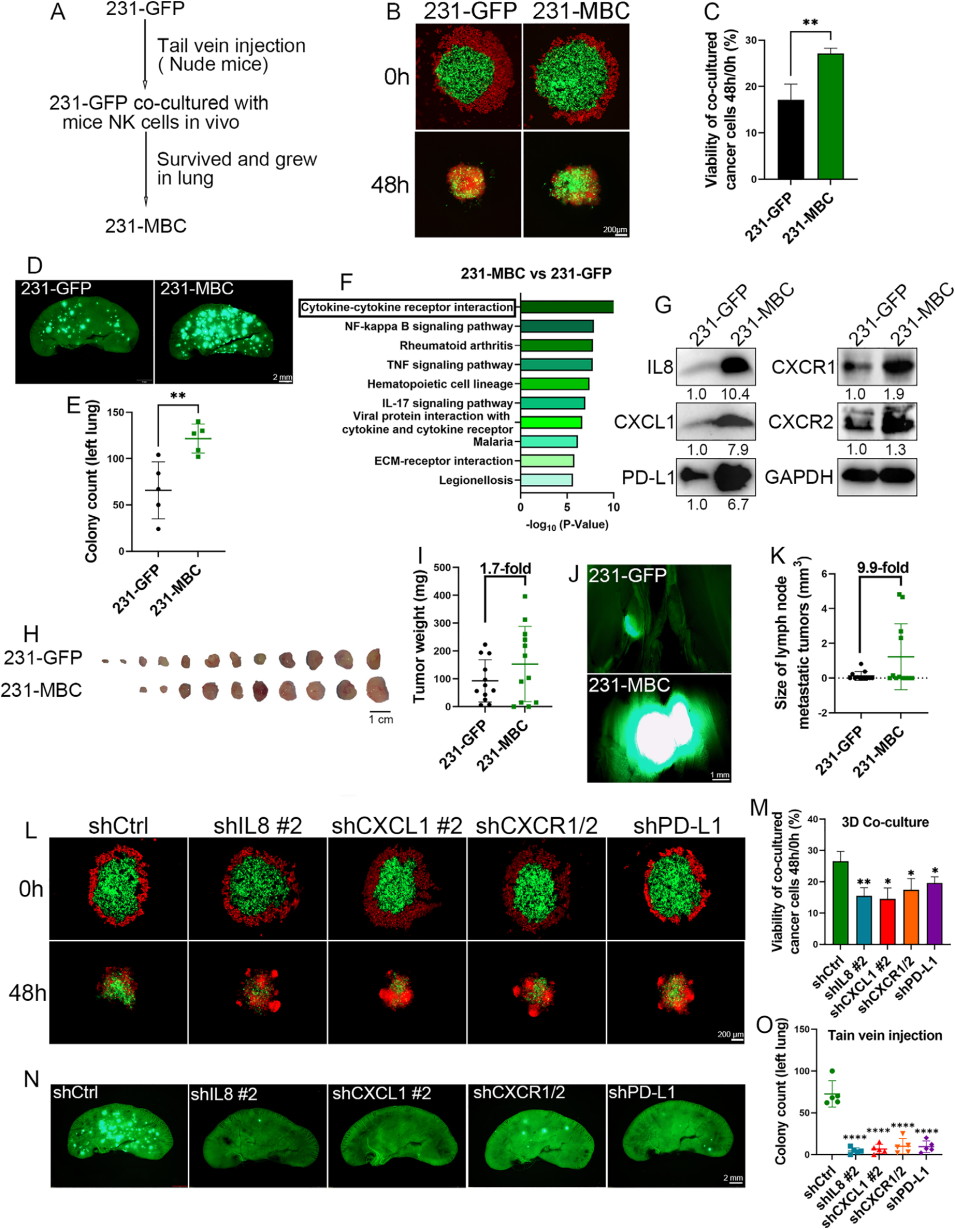
Metastatic TNBC cells rely on the IL8/CXCL1−CXCR1/2 signaling. (A) Workflow for illustrating the generation of metastatic 231‐MBC cells. (B, C) Representative images and quantification of 3D co‐culture of 231‐GFP or 231‐MBC cells with NK‐tdT cells at a ratio of 1:1 for 48 h. Scale bar, 200 µm. (D, E) Representative images and quantification of lung colonization through tail vein injection in nude mice after four weeks (*n* = 5 mice per group). Scale bar, 2 mm. (F, G) RNA‐seq analysis and Western blotting confirmed the upregulation of IL8, CXCL1, CXCR1/2, and PD‐L1. (H) Representative images of primary tumors 42 days after implantation of cancer cells into the mammary fat pad of nude mice (*n* = 6 mice per group). Scale bar, 1 cm. (I) Quantification of primary tumor volume. (J) Representative images of iliac lymph node metastatic tumors taken on day 42. Scale bar, 1 mm. (K) Quantification of iliac lymph node metastatic tumors. (L, M) Representative images and quantification of NK cell co‐culture with 231‐MBC knockdown cells in 3D conditions. Scale bar, 200 µm. (N, O) Representative images and quantification of lung metastatic colonies of 231‐MBC knockdown cells in nude mice (*n* = 5 mice per group). Scale bar, 2 mm. The results represent the means ± SD from three independent experiments. Significant differences were determined by Student's t‐test and one‐way ANOVA. *p < 0.05, **p < 0.01, ****p < 0.0001.

Metastatic breast cancer often exhibits higher metastatic potential than primary tumors, in part due to increased resistance to NK cell‐mediated cytotoxicity. To compare the changes in NK cell resistance before and after metastasis, we intravenously injected 231‐GFP cells into nude mice and isolated metastatic breast cancer cells (231‐MBC) from the lungs on day 28 post‐injection (Figure 5A). Through 3D co‐culture at a cancer‐to‐NK cell ratio of 1:1 for 48 h, 231‐MBC cells demonstrated 58% greater resistance to NK cell cytotoxicity than 231‐GFP cells (Figure 5B,C), consistent with previous observations that metastatic breast cells have higher resistance to NK cell‐mediated cytotoxicity [43].

Next, we compared in vitro tumorigenic properties between 231‐GFP and 231‐MBC cells. Although no differences were observed from the quantified results of MTT assay and sphere formation assay (Figure S7A–C), 231‐MBC cells formed approximately 1.4‐fold more colonies and exhibited 1.6‐fold higher migratory capacity compared to 231‐GFP cells (Figure S7D–G). We further compared the metastatic potentials between these two cell lines by injecting the cells into the tail‐vein of nude mice. Four weeks post injection, 231‐MBC cells formed 1.8‐fold more lung colonies than 231‐GFP cells did (Figure 5D,E). We also compared the tumor growth and metastatic abilities between these two cell lines using an orthotopic tumor model. The animal results showed that although tumor weight and lymph node metastasis did not differ statistically between 231‐GFP and 231‐MBC cells, the tumors in the 231‐MBC group were 1.7‐fold larger in size and 9.9‐fold bigger in the lymph node metastatic area compared to the 231‐GFP group. These findings suggest that 231‐MBC cells may have a stronger ability to resist NK cells in the lymphatic system, resulting in the formation of much bigger metastatic tumors in the lymph nodes (Figure 5H–K).

Through RNA‐seq and KEGG pathway analyses, we found that the “cytokine‐cytokine receptor interaction” pathway was also enriched in 231‐MBC cells (Figure 5F). Western blot analysis further showed that the protein levels of IL8, CXCL1, their corresponding receptors of CXCR1 and CXCR2, and PD‐L1 were upregulated in 231‐MBC cells (Figure 5G), suggesting the involvement of IL8/CXCL1−CXCR1/2−PD‐L1 signaling axis in the enhanced resistance to NK cell‐mediated cytotoxicity.

To validate the role of this pathway in 231‐MBC cells, we knocked down IL8, CXCL1, CXCR1/2, or PD‐L1 in 231‐MBC cells. All these genes were successfully knocked down in 231‐MBC cells (Figure S8A). Knockdown of IL8, CXCL1, or CXCR1/2 significantly reduced colony formation ability by 49%–59% and reduced the abilities of cell migration by 63%–74% (Figure S8B–E), suggesting that the IL8/CXCL1−CXCR1/2 signaling pathway contributes to the promotion of clonogenicity and cell motility. Moreover, knockdown of IL8, CXCL1, CXCR1/2, or PD‐L1 significantly reduced the viability of 231‐MBC cells against NK cell‐mediated killing in both 2D and 3D co‐culture models (Figure 5L,M and Figure S8F,G). Knockdown of IL8, CXCL1, and CXCR1/2 also diminished the inhibitory effect of 231‐MBC cells on NK cells, resulting in 1.5‐ to 2‐fold increases in NK cell viability in the conditioned medium (CM) of 231‐MBC cells (Figure S8H,I).

To test if other ligands compensate for CXCL1/IL8−CXCR1/2 axis suppression, we knocked down IL8, CXCL1, or CXCR1/2 and measured mRNA levels of CXCL1‐8 by qPCR in 231‐MBC and BT549‐clover cells. Although knockdown significantly decreased CXCL2 in 231‐MBC cells and both CXCL2 and CXCL3 in BT549‐clover cells (Figure S9A,B), mRNA levels of CXCL2 and CXCL3 were lower in TNBC tumors compared to normal breast tissues (Figure 2G and Figure S4B). Notably, disrupting any component coordinately downregulated both IL8 and CXCL1 in all cells, indicating their close coregulation and primary role in activating CXCR1/2 signaling.

Subsequently, we intravenously injected the knockdown cells into nude mice and observed the reduced lung metastasis of 231‐MBC cells by 94% (shIL8), 91% (shCXCL1), 86% (shCXCR1/2), and 87% (shPD‐L1), respectively (Figure 5N,O). Overall, these findings indicate that the enhanced metastatic potential of 231‐MBC cells is not attributable to increased proliferation, but rather to a combination of clonogenic, migratory, and NK cell evasive capabilities mediated via the IL8/CXCL1−CXCR1/2 signaling pathway.

### 2.6 Co‐Culture Induces ROS Elevation and Activates the ERK/JNK−AP‐1, JAK−STAT1 and NF‐κB Signaling Pathways to Upregulate the IL8, CXCL1 and PD‐L1 Expression

**FIGURE 6:**
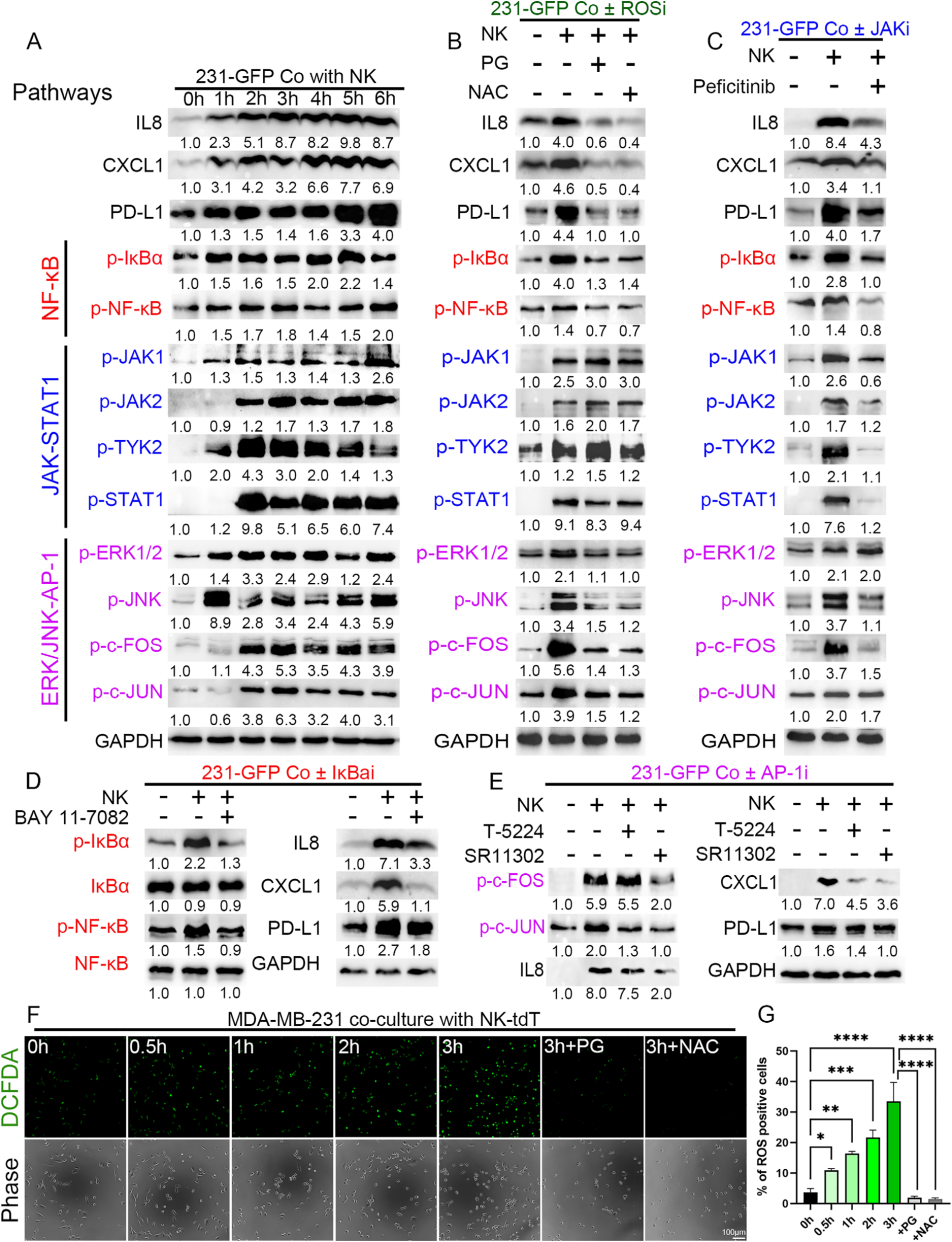
NK cell co‐culture induced ROS and activated NF‐κB, JAK‐STAT1, and ERK/JNK‐AP‐1 signaling pathways in 231‐GFP cells. (A) Time‐course Western blot analysis of IL8/CXCL1/PD‐L1 expression and signaling protein phosphorylation. (B–E) Western blot analysis of IL8/CXCL1/PD‐L1 expression and signaling protein phosphorylation in 231‐GFP cells mono‐ or co‐cultured with NK cells at a ratio of 2:1 for 6 h, with or without ROS inhibitor: PG (20 µm) and NAC (5 mm) (B), JAK inhibitor Peficitinib (10 µm) (C), IκBα inhibitor BAY 11–7082 (10 µm) (D), or AP‐1 inhibitors of T‐5224 (10 µm) and SR11302 (10 µm) (E). (F, G) ROS accumulation in MDA‐MB‐231 cells was measured by using a ROS detecting agent (DCFDA) for 3 h, with or without pretreatment with PG (20 µm) and NAC (5 mm). Scale bar, 100 µm. Fluorescence images are presented in the upper panels, and phase‐contrast images are shown in the lower panels. The results represent the means ± SD from three independent experiments. Significant differences were determined by one‐way ANOVA or the Student's t‐test *p < 0.05, **p < 0.01, ***p < 0.001, ****p < 0.0001.

It has been reported that the upstream regulatory factors of IL8, CXCL1, and PD‐L1 involve the JAK−STAT, NF‐κB, and ERK/JNK−AP‐1 signaling pathways [44, 45, 46]. To explore the specific mechanisms underlying the upregulation of IL8, CXCL1, and PD‐L1 under the co‐culture conditions, we co‐cultured 231‐GFP cells with NK cells at a cancer‐to‐NK cell ratio of 2:1 for 6 h (Figure 6A). After 1 h of co‐culture, the expression levels of IL8, CXCL1, and PD‐L1 had already increased by 2.3‐fold, 3.1‐fold, and 1.3‐fold, respectively. This was accompanied by elevating the levels of phosphorylated nuclear factor kappa‐light‐chain‐enhancer of activated B cells (p‐NF‐κB), phosphorylated nuclear factor of kappa light polypeptide gene enhancer in B‐cells inhibitor alpha (p‐IκBα), phosphorylated Janus kinase 1 (p‐JAK1), phosphorylated tyrosine kinase 2 (p‐TYK2), phosphorylated extracellular signal‐regulated protein kinases 1 and 2 (p‐ERK1/2), and phosphorylated c‐Jun N‐terminal kinases (p‐JNK), indicating activation of NF‐κB, JAK‐STAT, and ERK/JNK pathways. By 2 h, phosphorylation of Janus kinase 2 (JAK2), signal transducer and activator of transcription 1 (STAT1), c‐FOS, and c‐JUN was detected (c‐FOS and c‐JUN could form a heterodimer named AP‐1). By 4 h, IL8, CXCL1, and PD‐L1 expression were further upregulated to 8.2‐fold, 6.6‐fold and 1.6‐fold, respectively. These results suggest that p‐NF‐κB initiates transcription of these genes as early as 1 h, while subsequent JAK−STAT1 and ERK/JNK−AP‐1 activation further elevated their expression (Figure 6A and Figure S10A). Similar signaling activation was confirmed in BT549‐clover cells (Figure S10E).

Since both ERK1/2 and NF‐κB phosphorylation can be modulated by reactive oxygen species (ROS), and IFN‐γ can induce ROS in cancer cells, we hypothesized that ROS may be the upstream regulator of NF‐κB and ERK/JNK signaling pathways [39, 47]. To test this hypothesis, we measured ROS levels during co‐culture experiments and observed an approximately three‐fold increase at 0.5 h and rising progressively in MDA‐MB‐231 (Figure 6F,G). This suggests that ROS upregulation may facilitate phosphorylation and subsequent activation of NF‐κB and ERK/JNK signaling pathways. To investigate this further, we pretreated 231‐GFP cells with ROS inhibitors, specifically propyl gallate (PG) and N‐acetyl‐cysteine (NAC), followed by co‐culture with NK cells. Pretreatment with ROS inhibitors suppressed upregulation of IL8, CXCL1, and PD‐L1, and reduced levels of p‐IκBα, p‐NF‐κB, p‐ERK1/2, p‐JNK, p‐c‐FOS, and p‐c‐JUN. In contrast, the enhancement of p‐JAK1, p‐JAK2, p‐TYK2, and p‐STAT1 was not affected, indicating ROS‐independent activation of JAK−STAT1 signaling (Figure 6B and Figure S10B).

We next used inhibitors targeting JAK (Peficitinib), STAT1 (Fludarabine), IκBα (BAY 11–7082), and AP‐1 (T‐5224, SR11302) in 231‐GFP cells with or without NK cell co‐culture. The results showed that Peficitinib prevented NK‐induced upregulation of IL8, CXCL1, and PD‐L1, and suppressed phosphorylation of STAT1, IκBα, NF‐κB, JNK, and c‐FOS, suggesting that JAK activation influences IL8, CXCL1, and PD‐L1 expression both directly via STAT1 and indirectly through NF‐κB and ERK/JNK pathways (Figure 6C and Figure S10C).

Moreover, treatment with the STAT1 inhibitor (Fludarabine), IκBα inhibitor (BAY 11–7082), and AP‐1 inhibitor (T‐5224, SR11302) suppressed the expression of IL8, CXCL1, and PD‐L1 during co‐culture, confirming that JAK−STAT1, NF‐κB, and ERK/JNK−AP‐1 pathways contribute to the upregulation of these signaling molecules. Specifically, PD‐L1 upregulation is predominantly regulated by the JAK−STAT1 signaling pathway, CXCL1 by the NF‐κB signaling pathway, and IL8 by the ERK/JNK−AP‐1 signaling pathway (Figure 6D,E and Figure S10D).

### 2.7 IL8/CXCL1 Bind to CXCR1/2 Receptors to Inhibit NK Cell Function by STAT3 Phosphorylation and Promote Cancer Cells Metastasis via AKT−BCL‐2 Signaling Axis

**FIGURE 7:**
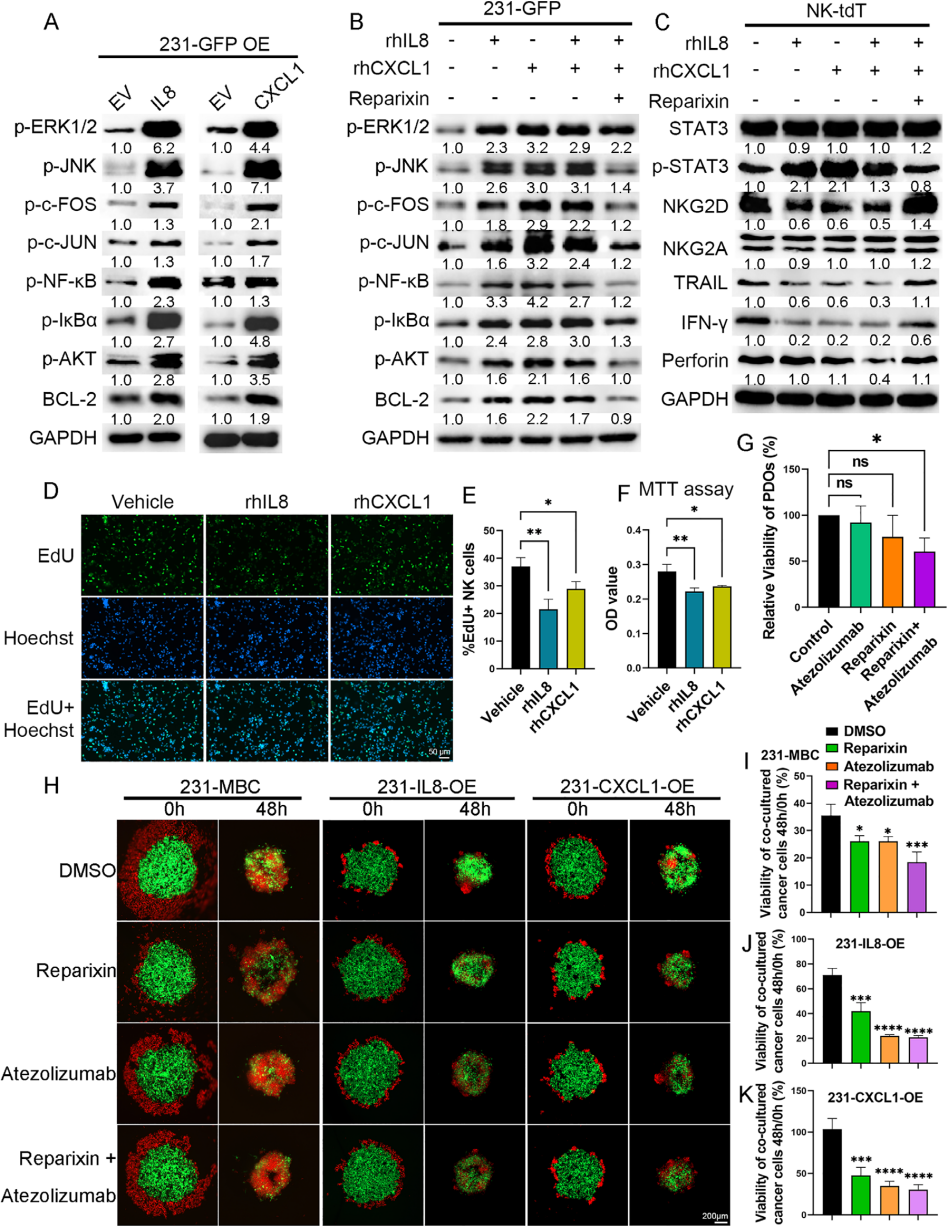
IL8 and CXCL1 enhanced TNBC cell survival and suppressed NK cell function via CXCR1/2. (A, B) Overexpression (A) or treatment with or without recombinant proteins (B) of IL8/CXCL1 activate ERK/JNK, NF‐κB, AKT−BCL‐2 signaling in cancer cells: rhIL8 (100 ng/mL), rhCXCL1 (100 ng/mL), and CXCR1/2 inhibitor Reparixin (10 µm) for 6 h. (C) Western blot analysis of STAT3 activation and NK effector molecule expression after treatment with DMSO, rhIL8 (100 ng/mL), rhCXCL1 (100 ng/mL), and CXCR1/2 inhibitor Reparixin (10 µm) for 6 h. (D, E) Representative images and quantification of NK cell proliferation after treatment with rhIL8 (100 ng/mL) and rhCXCL1 (100 ng/mL) for 12 h using EdU incorporation assay. Scale bar, 50 µm. (F) NK cell proliferation was measured by MTT assay after treatment with rhIL8 (100 ng/mL) and rhCXCL1 (100 ng/mL) for 48 h. (G) Combined treatment with Atezolizumab and Reparixin significantly suppressed the growth of human TNBC patient‐derived organoids ex vivo. (H–K) Representative images and quantification of NK‐tdT cell co‐cultured with 231‐MBC cells (NK:cancer = 1:1), 231‐IL8‐OE or 231‐CXCL1‐OE cells (NK:cancer = 1:2) for 48 h with the treatment of CXCR1/2 inhibitor (Reparixin, 10 µm), anti‐PD‐L1 monoclonal antibody (Atezolizumab, 20 µg/mL), or in combination. Scale bar, 200 µm. The results represent the means ± SD from three independent experiments. Significant differences were determined by one‐way ANOVA. *p < 0.05, **p < 0.01, ***p < 0.001, ****p < 0.0001, ns, not significant.

To further explore the effects of upregulated IL8 and CXCL1 on tumor cells and NK cells, IL8 and CXCL1 were overexpressed in 231‐GFP cells. Compared with control cells, the levels of p‐ERK1/2, p‐JNK, p‐c‐FOS, p‐c‐JUN, p‐NF‐κB, p‐IκBα, p‐AKT, and BCL‐2 were elevated in the overexpressed cells, indicating enhanced ERK/JNK−AP‐1, NF‐κB, and AKT signaling along with increased anti‐apoptotic and metastatic potential (Figure 7A).

Subsequently, human recombinant IL8 (rhIL8) and CXCL1 (rhCXCL1) proteins were added to the culture medium of 231‐GFP cells and the culture medium of NK‐tdT cells to evaluate the effects of these two cytokines on tumor cells and NK cells. Both cytokines, alone or combined, activated ERK/JNK−AP‐1 and NF‐κB pathways by upregulating p‐ERK1/2, p‐JNK, p‐c‐FOS, p‐c‐JUN, p‐NF‐κB, and p‐IκBα in 231‐GFP cells (Figure 7B). The addition of rhIL8 and rhCXCL1 also increased p‐AKT and BCL‐2 by 1.6‐ to 2.2‐fold, suggesting an enhancement of the anti‐apoptotic capacity in 231‐GFP cells. Notably, the upregulation of these signaling molecules was attenuated following treatment with Reparixin, a selective inhibitor of CXCR1/2, suggesting that the CXCR1/2 receptors play a critical role in mediating the functional activities of rhIL8 and rhCXCL1 in 231‐GFP cells (Figure 7B).

In NK‐tdT cells, rhIL8 and rhCXCL1 induced STAT3 phosphorylation but reduced NKG2D, tumor necrosis factor‐related apoptosis‐inducing ligand (TRAIL), and IFN‐γ expression. Simultaneous administration of rhIL8 and rhCXCL1 was associated with reduced perforin protein levels, while NKG2A expression remained unchanged. These effects could also be attenuated through the application of CXCR1/2 inhibitor (Reparixin), indicating that rhIL8 and rhCXCL1 may suppress NK cells’ function via the CXCR1/2 receptors (Figure 7C).

Next, we investigated the effects of rhIL8 and rhCXCL1 on NK cells’ proliferation using EdU and MTT assays. Treatment with 100 ng/mL of rhIL8 and rhCXCL1 decreased the EdU‐positive rate of NK cells by 41% and 22%, respectively (Figure 7D,E). MTT assay also demonstrated that treatment with rhIL8 and rhCXCL1 reduced NK cell proliferation by 20.8% and 15.6%, respectively (Figure 7F), confirming that both cytokines could inhibit NK cell proliferation. Collectively, these findings demonstrate that IL8/CXCL1 signaling through CXCR1/2 suppresses NK cell effector function and proliferation, while simultaneously enhancing TNBC cell survival via activation of ERK/JNK−AP‐1, NF‑κB, and AKT−BCL‐2 pathways.

### 2.8 Combined Treatment of Reparixin With Atezolizumab Significantly Enhanced NK Cell Cytotoxicity Against TNBC Patients

To assess potential therapeutic strategies, we co‐cultured 231‐MBC, 231‐IL8‐OE, and 231‐CXCL1‐OE cells with NK cells in the presence of Reparixin, anti‐PD‐L1 monoclonal antibody (Atezolizumab), or a combination of both. Although both monotherapies decreased the viability of cancer cells against NK‐mediated cytotoxicity, the combined treatment further significantly reduced tumor cell viability by 48% in 231‐MBC cells, 70% in 231‐IL8‐OE cells, and 71% in 231‐CXCL1‐OE cells (Figure 7H–K). In addition, combinational treatment with Reparixin and Atezolizumab significantly inhibited the growth of TNBC patient‐derived organoids (Figure 7G), supporting the translational applicability of this strategy in treating patients with TNBC.

**FIGURE 8:**
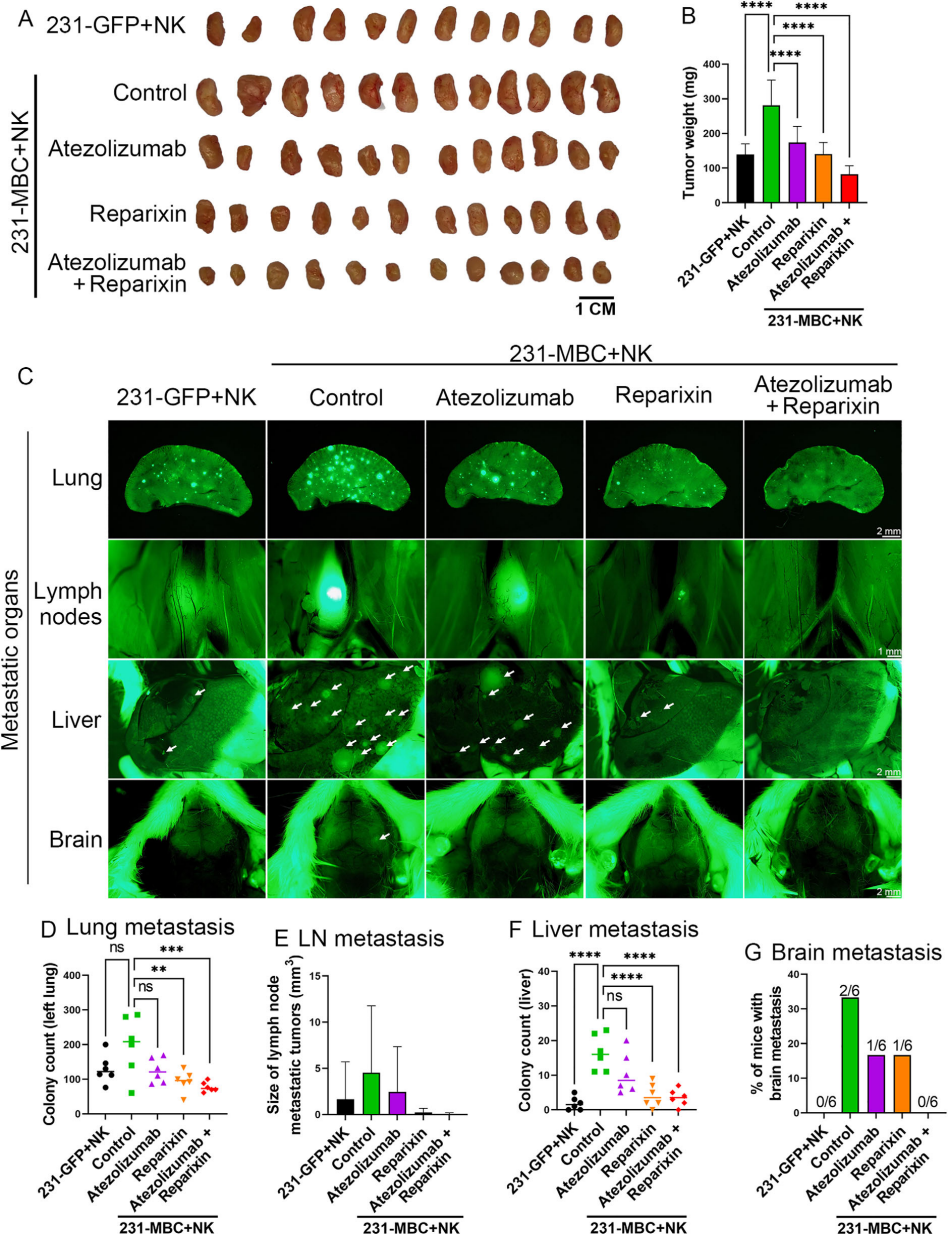
Combination therapy with Atezolizumab and Reparixin reduced tumor growth and metastasis in orthotopic breast cancer models. (A, B) Representative images and quantification of orthotopic primary tumor growth. NSG mice bearing tumors derived from co‐cultured 231‐GFP or 231‐MBC cells were treated for three weeks with Atezolizumab (2.5 mg/kg, twice weekly), Reparixin (20 mg/kg/day for 10 days), or the combination of both agents (*n* = 6 per group). Scale bar, 1 cm. (C) Representative images of metastatic lesions in the indicated organs. Scale bars: 2 mm (lung, liver, brain) or 1 mm (lymph nodes). (D–G) Quantification of metastatic nodules in the lung (D), iliac lymph nodes (E), liver (F), and brain (G). The results represent the means ± SD from three independent experiments. Significant differences were determined by one‐way ANOVA. **p < 0.01, ***p < 0.001, ****p < 0.0001. ns, not significant.

To determine the therapeutic effects of inhibiting the CXCL1/IL8 and PD‐L1‐activated signaling pathway in reducing tumor growth and metastasis in vivo, we co‐cultured metastatic TNBC 231‐MBC cells with NK cells for 24 h and injected them into NSG (NOD scid gamma) mice, which are severely immunodeficient due to the lack of functional T cells, B cells, and NK cells. One week after tumor establishment, mice received Atezolizumab (2.5 mg/kg, twice weekly), Reparixin (20 mg/kg/day for 10 days), or a combination of both treatments for up to three weeks. At the end of animal experiments, the mice in the control group developed rapid progressive disease characterized by a large tumor burden (>10 mm), body‐weight loss, and marked deterioration of physical conditions. In contrast, the mice in the drug treatment groups did not show any visible altered physical conditions. Individual administration of Atezolizumab or Reparixin significantly reduced tumor weight by 38.3% and 50.1%, while combinational treatment achieved the strongest response with a 70.7% reduction of tumor weight (Figure 8A,B).

More importantly, control mice developed extensive late‑stage, multi‑organ metastases, including lung, lymph nodes, liver, and brain, indicating late‐stage, near‐lethal disease. In contrast, mice treated with CXCR1/2 inhibitor displayed significantly fewer metastatic tumors in the lung, lymph nodes, liver, and brain than the control mice. Although administration of anti‐PD‐L1 antibody alone did not significantly reduce distant metastasis, the combinational therapy of using both anti‐PD‐L1 antibody and CXCR1/2 inhibitor achieved the best anti‐metastatic effects by greatly reducing metastatic tumors to their minimum number in the lung, lymph nodes, and liver and abolishing brain metastasis (Figure 8C–G).

To further support the therapeutic relevance of inhibiting PD‐L1 and CXCR1/2, we also performed an in vivo experiment by using a tail‐vein lung metastasis model, in which double knockdown of CXCR1/2 and PD‐L1 in 231‐MBC cells resulted in a significant reduction of lung metastatic colony formation than the control group (Figure S11A,B).

Taken together, these complementary in vivo and patient‐derived models demonstrate that simultaneous blockade of CXCR1/2 and PD‐L1 produces a profound delay in lethal metastatic progression, providing strong evidence for a survival benefit of the combinatorial therapy in treating metastatic TNBC.

### 2.9 High Expression of IL8 and CXCL1 Correlates With Reduced NK Cell Infiltration, Increased Tumor Malignancy, and Shorter Distant Metastasis‐Free Survival (DMFS) in Breast Cancer Patients

**FIGURE 9:**
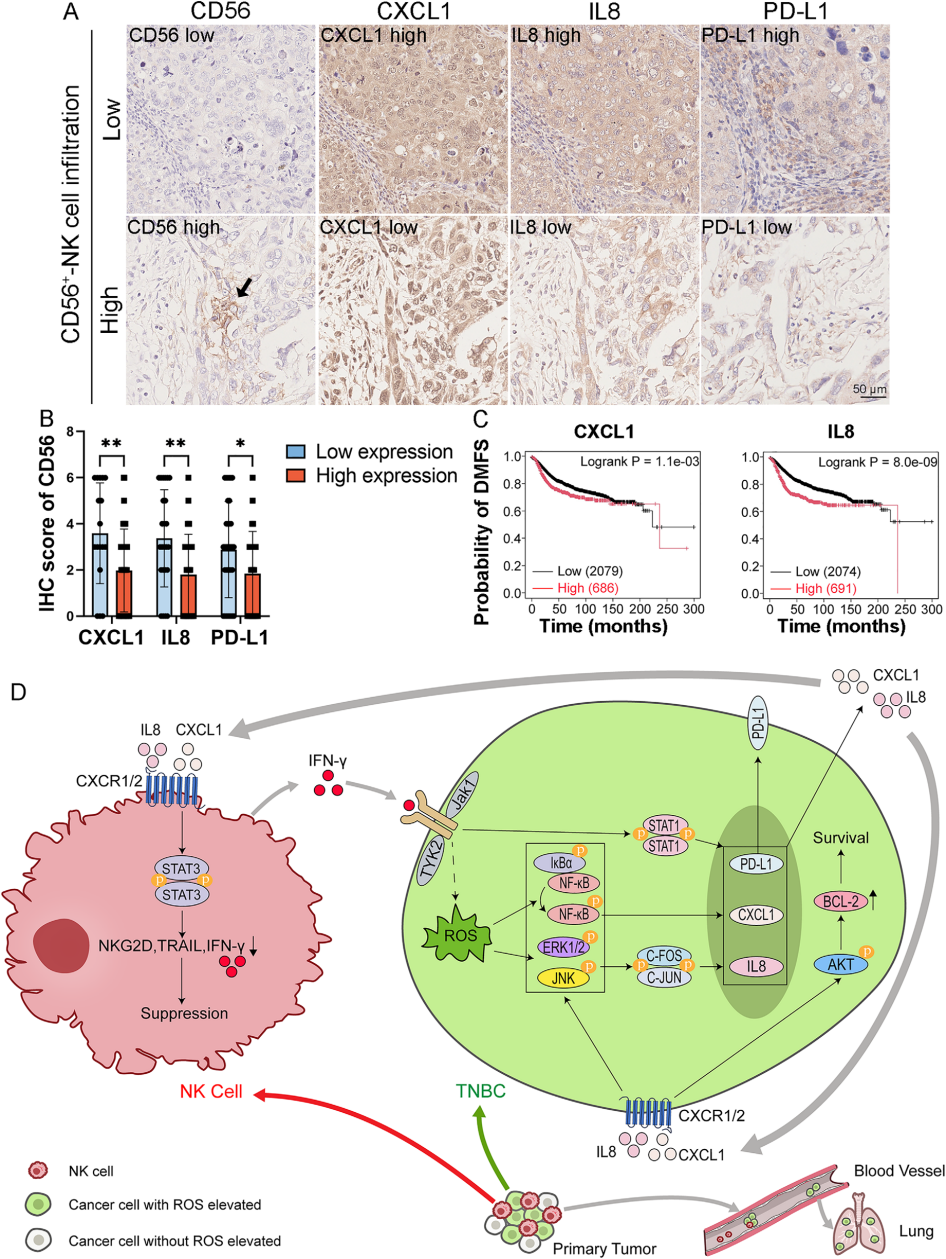
High IL8 and CXCL1 expression correlated with less NK cell infiltration and poor patient outcome. (A) Representative IHC images of NK cell marker CD56, IL8, CXCL1, and PD‐L1 in human TNBC tissue (*n* = 60). Scale bar, 50 µm. (B) Negative correlation between the IHC scores of IL8 and CXCL1 staining and NK cell infiltration, assessed by CD56 expression, in TNBC patients (*n* = 60). (C) Correlation between the expression of IL8, CXCL1, and distant metastasis‐free survival (DMFS) in TNBC patients analyzed from the Kaplan Meier plotter database (*n* = 2765). (D) Schematic diagram showing how TNBC cells utilize IL8 and CXCL1 to suppress NK cells’ function, promote cancer cell survival, and metastasis. Significant differences were determined by Student's *t*‐test. *p < 0.05, **p < 0.01.

To evaluate the correlation between IL8/CXCL1 expression and NK cell infiltration in TNBC patients, we performed IHC staining on tissues from 60 TNBC patients assembled in a tissue microarray provided by Outdo Biotech Company. We calculated the IHC score by combining the proportion score and the intensity score as previously described [48]. High expression was defined based on the median values of the IHC results: IHC scores of 7–8 for IL8 and CXCL1, 6–8 for PD‐L1, 4–6 for CD56, and a Ki‐67 index of ≥50%. Representative images and quantitative analyses revealed that TNBC tumors with higher levels of CXCL1/IL8 and PD‐L1 exhibited significantly lower levels of CD56^+^‐NK cell infiltration into the tumor tissues (Figure 9A,B). Further analysis of IHC‐stained clinical samples revealed that in TNBC tissues with high IL8/CXCL1 expression, the number of low CD56 tissues was 4.0–5.3‐fold higher than that of high CD56 tissues, regardless of PD‐L1 expression (Figure S11C). This observation indicates that IL8/CXCL1‐mediated NK cell suppression may present in primary tumors of TNBC patients, which is independent of PD‐L1 expression in tumor tissue.

Since Ki‐67 is an established marker of tumor proliferation and aggressiveness [49], we further assessed the correlation between Ki‐67 index provided by Outdo Biotech Company and IL8/CXCL1 expression measured by IHC analysis on tissue microarray. The results showed that tumors with high expression of IL8 or CXCL1 displayed significantly higher index of Ki‐67, while no significant correlations were observed between PD‐L1 and Ki‐67 (Figure S11D). More importantly, the high expression of CXCL1 and IL8 was associated with shorter distant metastasis‐free survival (DMFS) (Figure 9C). These results suggest that IL8 and CXCL1 correlate with reduced NK cell infiltration, increased malignancy, and earlier metastasis in TNBC patients.

The findings of this study indicate that during the co‐culture of TNBC cells with NK cells, NK‐derived IFN‐γ induces PD‐L1, IL8, and CXCL1 expression via JAK−STAT1 signaling and stimulates ROS production. Elevated ROS levels subsequently activate the NF‐κB and ERK/JNK−AP‐1 signaling pathways, resulting in upregulation of CXCL1 and IL8. PD‐L1 binding to PD‐1 on NK cells induces the programmed cell death of NK cells, leading to the reduction of their cytotoxic effect on cancer cells. The secreted IL8 and CXCL1 can also act in an autocrine or paracrine manner on CXCR1/2 receptors of TNBC cells, further amplifying IL8, CXCL1, and PD‐L1 expression and enhancing BCL‐2‐mediated survival. On the other hand, these cytokines can suppress the expression of NKG2D, TRAIL, and IFN‐γ in NK cells, thereby impairing their cytotoxic activity (Figure 9D). In summary, TNBC cells utilize IL8 and CXCL1 to suppress NK cells’ function, thereby facilitating their own survival, immune evasion, and metastatic potential.

## 3 Discussion

TNBC represents the most aggressive subtype of breast cancer, characterized by high metastatic potential and limited therapeutic options, with the mesenchymal‐like subtype having the worst prognosis in patients [38, 50]. Although NK cells represent a promising immunotherapeutic approach due to their innate ability to eliminate malignant cells, their efficacy against TNBC remains unsatisfactory [51, 52, 53]. Here we showed that TNBC cells, especially mesenchymal‐like subtypes, were more resistant to NK cell‐mediated cytotoxicity than non‐TNBC cells. Transcriptomic and functional analyses identified IL8, CXCL1, and PD‐L1 as key mediators, revealing a previously unrecognized signaling axis for immune evasion and metastasis.

To address potential redundancy among CXCR1/2 ligands, we profiled ELR+ chemokines in TNBC models. Our data indicate that CXCL1 and IL8 constitute the dominant tumor‐derived CXCR1/2 ligands in TNBC models. These chemokines are highly expressed in TNBC cells, selectively suppressed upon axis knockdown, and do not exhibit compensatory replacement by other ELR+ chemokines. These findings suggest that, despite the presence of multiple immune‐evasive pathways, the IL8/CXCL1‐CXCR1/2 axis represents a tumor‐specific and functionally dominant chemokine program enabling TNBC cells to resist the damage of NK cells.

Mechanistically, co‐culture of TNBC cells with NK cells rapidly upregulated IL8 and CXCL1 expression via ROS‐dependent activation of the ERK/JNK−AP‐1 or NF‐κB pathways, followed by sustained amplification via a CXCL1/IL8−CXCR1/2 autocrine loop. These cytokines enhanced tumor survival through AKT−BCL‐2 anti‐apoptotic pathway, but also directly suppressed NK cell function by downregulating NKG2D, TRAIL, and IFN‐γ expression. Moreover, PD‐L1 emerged as a downstream target of IFN‐γ−JAK−STAT1 and IL8/CXCL1−CXCR1/2 signaling, which further contributed to immune suppression of NK cells. These findings were consistent across mesenchymal‐like TNBC cell lines and were validated in vivo, where modulation of IL8, CXCL1, or PD‐L1 significantly altered tumor growth and metastasis in mouse models.

Our results are consistent with previous studies linking IL8 and CXCL1 to poor prognosis and immune suppression in breast cancer patients [54, 55, 56]. For instance, IL8 has been shown to promote EMT and recruitment of immunosuppressive cells [57], while CXCL1 enhances angiogenesis and metastasis [58]. However, we extend these findings by demonstrating that close interactions between NK cells and TNBC cells trigger IL8 and CXCL1 expression in TNBC cells, establishing a feed‐forward loop that amplifies both immune evasion and metastasis. We also provide mechanistic evidence that ROS act as early upstream signaling molecules, linking NK cell‐derived IFN‐γ to the activation of ERK/JNK−AP‐1 and NF‐κB pathways.

Previous studies showed that macrophage‐derived IL8 and CXCL1 induced PD‐L1 expression in cancer cells [59, 60]. We further identified a positive feedback loop among IL8, CXCL1, and PD‐L1 in TNBC cells, wherein each factor reinforces the others. This triad cooperatively enhances TNBC cell survival and suppresses NK cell activity, suggesting that combined targeting of these molecules may yield synergistic therapeutic benefits. Consistently, we showed that CXCR1/2 inhibition (Reparixin) combined with anti‐PD‐L1 therapy (Atezolizumab) significantly enhanced NK cell cytotoxicity against TNBC cells. The combination of Reparixin and Atezolizumab markedly reduced tumor burden in the mouse model and inhibited the growth of TNBC patient‐derived organoids. Moreover, metastatic TNBC cells (231‐MBC) exhibit heightened dependence on the IL8/CXCL1−CXCR1/2−PD‐L1 signaling pathway, underscoring its role in advanced disease. These findings are supported by clinical data showing that high IL8/CXCL1 expression correlates with reduced NK cell infiltration, increased Ki‐67 positivity, and shorter distant metastasis‐free survival in TNBC patients.

We noticed that the clinical trials of CXCR1/2 inhibitor reparixin (No. NCT02370238) and anti‐PD‐L1 antibody atezolizumab (IMpassion131 trial) failed to confirm a survival benefit in breast cancer patients [61, 62]. Based on our mechanistic findings, we propose that this may be suboptimal. Specifically, our study shows that the IL8/CXCL1–CXCR1/2–PD‐L1 axis not only directly suppresses NK cell function but also upregulates PD‐L1 expression in TNBC cells, which could in principle inhibit T cell‑mediated immunity as well. Therefore, combining a CXCR1/2 inhibitor with chemotherapy alone might only partially relieve NK cell suppression while leaving the PD‑L1‑mediated T cell suppression intact. A more rational strategy is to combine CXCR1/2 inhibition with PD‑1/PD‑L1 blockade to simultaneously restore both innate and adaptive antitumor immunity. This hypothesis has been validated in a murine model of pancreatic cancer and in patients with metastatic melanoma (clinical trial information: NCT03161431), and warrants further testing in preclinical TNBC models and clinical trials [63].

It is important to note that the primary TNBC model used in this study is MDA‐MB‐231 cells, which have a high basal expression of PD‐L1. Given that only a subset (15%–30%) of primary TNBC tumors express PD‐L1, we validated our findings in TNBC tumors that express low levels of PD‐L1 and TNBC cells with a minimum level of PD‐L1. The data confirm that the IL8/CXCL1−CXCR1/2 axis can drive the resistance of NK cells in a manner that is both applicable to PD‐L1‐low and ‐high TNBC cells, highlighting its broad therapeutic potential across TNBC subtypes with different levels of PD‐L1.

However, this study also has certain limitations. NK‐92MI cells, as the main experimental cell line in this study, exhibited stronger cytotoxicity than primary human NK cells. Future validation using primary human NK cells from multiple donors is essential to confirm the physiological relevance of our findings. Although we validated our core findings across multiple TNBC cell lines and patient‐derived organoids, we cannot exclude the possibility that some subtle mechanistic details may vary across the full spectrum of TNBC molecular subtypes. Future work in broader subtype‑specific and primary TNBC samples is warranted. In addition, the use of single inhibitor concentrations may raise concerns about specificity and off‑target effects. Future dose‑response studies are needed to establish optimal working concentrations.

In conclusion, we revealed that TNBC cells utilize IL8 and CXCL1 to subvert NK cell‐mediated immunity and enhance metastatic potential. These findings highlight the IL8/CXCL1−CXCR1/2 signaling axis as a promising therapeutic target, either alone or in combination with PD‐L1 blockade, for TNBC treatment. Furthermore, IL8 and CXCL1 expression may serve as prognostic markers to identify TNBC patients at high risk of metastasis who could benefit from intensified immunotherapy. Future studies should evaluate the translational potential of combining CXCR1/2 inhibitors with PD‐L1 blockade in clinical trials.

## 4 Experimental Section

### 4.1 Cell Lines and Cell Culture

Breast cancer cell lines (MCF‐7 (RRID: CVCL_0031), T47D (RRID: CVCL_0195), BT474 (RRID: CVCL_0179), SKBR3 (RRID: CVCL_0033), UACC893 (RRID: CVCL_1782), MDA‐MB‐231 (RRID: CVCL_0062), BT549 (RRID: CVCL_1092), MDA‐MB‐468 (RRID: CVCL_0419)) and human embryonic kidney 293T (RRID: CVCL_0063) cells were obtained from the American Type Culture Collection (ATCC). HCC1937 (RRID: CVCL_0290) breast cancer cells and NK‐92MI (RRID: CVCL_3755) natural killer cells were provided by Prof. Xiaoling Xu and Prof. Qi Zhao, respectively (Faculty of Health and Science, University of Macau, Macau, China). All RRID are from Cellosaurus. Breast cancer cell lines were transfected with plasmids encoding C3, GFP or clover as previously described [39]. 231‐MBC cell line was derived from 231‐GFP through isolation of lung metastatic colonies in nude mice by Dr. Renfei Wu. [64] NK‐92MI cells were stably transduced with tandem dimer Tomato (tdT) red fluorescent protein by Dr. Hongmei Yang (designated NK‐tdT). All cells were uncontaminated.

Cells were maintained at 37°C under 5% CO_2_ in the following media supplemented with 10% fetal bovine serum (FBS; #10270‐106, Gibco) and 1% penicillin‐streptomycin (#15140122, Thermo Fisher Scientific). T47D and BT549 cells were cultured in RPMI 1640 medium (#2174257, Thermo Fisher Scientific, USA). Other breast cancer cells and 293T cells were cultured in Dulbecco's modified Eagle's medium (DMEM, #12100046, Thermo Fisher Scientific, USA). NK‐tdT cells were cultured in α‐MEM (#12000‐022, Gibco) containing 12.5% FBS, 12.5% horse serum (#16050‐122, Gibco, USA), 36 mg/L myo‐Inositol (#I7508, Sigma, USA), 4.414 mg/L Folic acid (#F8758, Sigma, USA), and 3.9 µL /L 2‐Mercaptoethanol (#M3418, Sigma, USA).

### 4.2 Tumor Sphere Formation Assay

For the 3D co‐culture experiment, green fluorescent protein (GFP)‐labeled cancer cells (3000 cells/well) were co‐cultured with or without tdTomato‐labeled NK‐tdT cells in ultralow‐attachment round‐bottom plates (#7007, Corning) at a ratio of 2:1 or 1:1 (for 231‐MBC cells) for 48 h. Fluorescence images were acquired every 12 h using a Carl Zeiss Axio Observer Z1 Inverted Fluorescence Microscope. In the monoculture tumor growth assay, cancer cells (200 cells/well) were seeded into the plates and monitored weekly via microscopy. Total fluorescence intensity was quantified using ImageJ software to evaluate the cell viability following co‐culture.

### 4.3 Conditioned Medium Treatment

Cancer cells (2 × 10^6^) were seeded into the 10 cm culture dish and incubated with 10 mL complete growth medium for 24 h. Following incubation, the supernatants were collected and centrifuged at 300 × g for 5 min to obtain the conditioned medium. NK‐tdT cells (3000 cells/well) were seeded into ultralow‐attachment 96‐well plates containing 50 µL of complete α‐MEM per well. Subsequently, 150 µL of the conditioned medium was added to each well. Images were captured every 12 h after the cells settled, and cell viability was assessed by measuring the total tdTomato fluorescence intensity using ImageJ software.

### 4.4 2D Co‐Culture Assay

Cancer cells (1 × 10^4^) were seeded into flat‐bottom 96‐well plates and cultured overnight. Following attachment of the cancer cells, NK‐tdT cells were added to the culture plates according to the cancer‐to‐NK ratio. Fluorescence imaging was performed every 12 h for a total duration of 48 h using either a Carl Zeiss Axio Observer Z1 Inverted Fluorescence Microscope, or a Nikon TiE fluorescent microscope to capture whole‐well images of the 96‐well plates.

### 4.5 Calcein‐AM Assay

Cancer cells (1 × 10^4^ cells/well) were seeded in 100 µL per well on a flat‐bottom 96‐well plate. After attachment, the cancer cells were stained with calcein‐AM (2.5 µm) (#C3099, Thermo Fisher Scientific, USA) for 30 min at 37°C, then washed twice with phenol red‐free DMEM (#21063029, Gibco, USA). NK‐92MI cells were added at different cancer‐to‐NK ratios (1:2, 1:1, 2:1, 5:1), with a total volume of 200 µL. 1 h before the end of the experiment, 2 µL of Triton X‐100 (#T8787, Sigma‒Aldrich, USA) was added to certain well and incubated for 1 h to determine the maximum lysis values. After co‐culture for 6 h, the 96‐well plate was centrifuged, and 100 µL of the supernatant was collected for fluorescence measurement. Fluorescence intensity was measured by a Victor3 plate reader (PerkinElmer, UK) at an excitation wavelength of 485 nm and an emission wavelength of 535 nm. The percentage of specific lysis was calculated using the following formula:

$$\begin{array}{ccc} & & \mathrm{Specific}\;\mathrm{Lysis}\;\%\\ & & \;=\frac{\mathrm{Test}\;\mathrm{release}-\mathrm{Target}\;\mathrm{spontaneous}\;\mathrm{release}}{\mathrm{Maximun}\;\mathrm{release}-\mathrm{Target}\;\mathrm{spontaneous}\;\mathrm{release}}\times 100 \end{array}$$

### 4.6 RNA Sequencing Analysis

MCF7‐C3, 231‐GFP, and 231‐MBC cells were harvested and lysed using TRIzol reagent (#15596026, Thermo Fisher Scientific, USA). Total RNA was extracted and purified by Novogene (China) for RNA sequencing analysis. RNA quality was assessed by absorbance ratios (A260/A280 ≥ 1.8 and A260/A230 ≥ 1.8) and the RNA integrity number (RIN) value of 9 or higher was considered acceptable for sequencing. Differentially expressed genes were identified using a false discovery rate (FDR) < 0.05 and a |log2(fold change)| ≥ 1 as the statistical thresholds.

### 4.7 Reverse Transcription and Quantitative PCR (RT‐qPCR)

Total RNA was isolated using TRIzol reagent. Reverse transcription was carried out following the manufacturer's instructions for the iScript cDNA Synthesis Kit (#1778890, Bio‐Rad, USA). Quantitative PCR was performed using the iTaqTM Universal SYBR Green Supermix (#1725122, Bio‐Rad, USA) on the CFX96 TouchTM Real‐Time PCR Detection System (Bio‐Rad, USA). The relative gene expression levels were calculated using the ∆∆CT method, with GAPDH serving as the internal reference gene. The primer sequences used in this study are listed in Table S1.

### 4.8 Western Blot Analysis

Cells were harvested, washed with ice‐cold PBS, and centrifuged at 1500 rpm for 3 min. Pellets were resuspended with RIPA lysis buffer (150 mm NaCl, 50 mm Tris‐HCl, 0.5% SDS, and 1% Triton X‐100) supplemented with a protease inhibitor (#P8340, Sigma‒Aldrich, USA) and phosphatase inhibitor cocktail 2 and 3 (#P0044, #P5726, Sigma‒Aldrich, USA). Cell lysates were sonicated for 5 min, centrifuged at 14,000 rpm for 15 min (4°C). Supernatants were collected, and protein concentrations were determined using the Bradford assay (#5000006, Bio‐Rad, USA). Equivalent amounts of protein were separated by SDS‐PAGE and subsequently transferred onto nitrocellulose membranes (#1620112, Bio‐Rad, USA). Membranes were blocked with 5% blotting‐grade blocker (#1706404, Bio‐Rad, USA) and incubated with primary antibodies at 4°C overnight. After washing, the membranes were probed with HRP‐conjugated secondary antibodies for 1 h at room temperature. Protein bands were visualized using Clarity Western ECL Substrate (#1705060, Bio‐Rad, USA) and imaged using a ChemiDoc Touch System (#1708370, Bio‐Rad, USA). Antibody specifications are listed in Table S2.

### 4.9 Lung Colony Formation Assay

All mice were acclimatized and maintained under standard conditions in the Animal Facility of the University of Macau. All experimental procedures involving animals were conducted in accordance with the guidelines approved by the Animal Research Ethics Committee of the University of Macau (Approved Protocol ID: UMARE‐025‐2017 and UMARE‐026‐2017). 6‐ to 8‐week‐old female BALB/c athymic nude mice were intravenously injected with cancer cells (5 × 10^5^ cells/mouse) via the tail vein (*n* = 5 per group). After 28 days, the lungs were excised, and metastatic colonies were visualized using an Olympus MVX10 fluorescence microscope. The number of metastatic colonies in the left lung was quantified to assess the pulmonary metastatic potential of the cancer cells.

### 4.10 Orthotopic Model (Nude Mice, NOD/SCID Mice, and NSG Mice)

Female mice (6–8 weeks old) were anesthetized by intraperitoneal injection of 1.25% avertin, and cancer cells were orthotopically implanted into the fourth mammary fat pad. For nude mice, 2.5 × 10^6^ cancer cells were injected. For NOD/SCID and NSG models, cancer cells and NK‐tdT cells were pre‐co‐cultured at a 5:1 ratio for 24 h, followed by co‐injection of 2 × 10^6^ cancer cells and 4 × 10^5^ NK‐tdT cells in 50 µL PBS. In therapeutic studies using 231‐MBC cells in NSG mice, animals were randomized into four groups when tumor volumes reached ∼20 mm^3^ (∼7 days post‐implantation) and treated with vehicle control, Atezolizumab (2.5 mg/kg, i.v., twice weekly), Reparixin (20 mg/kg, i.p., daily for 10 days), or the combination. Body weight and tumor volume were monitored weekly, with tumor volume calculated as V = (L × W^2^)/2. Primary tumors were excised and weighed at 4–6 weeks post‐inoculation, and metastatic burden in the iliac lymph nodes and lungs was assessed using fluorescence stereomicroscopy (MVX10, Olympus). All animal experiments were approved by the Animal Ethics Committee of the University of Macau (protocol IDs: UMARE‐025‐2017 and UMARE‐026‐2017).

### 4.11 Lentivirus‐Delivered shRNA Knockdown and Gene Overexpression

All plasmids were purchased from the VectorBuilder company (Chicago, USA) and the sequences were selected based on the H‐b index calculation of the candidate molecules [65]. The target sequences of shRNA (Table S3) and the transcript ID of overexpression vectors (Table S4) are listed in the Supporting Information. The shRNA sequences were cloned into the pLV[shRNA]‐Bsd‐U6 or pLV[shRNA]‐Puro‐U6 lentiviral plasmid, while the overexpression vector constructs were cloned into the pLV[Exp]‐Bsd‐CMV+intron plasmid. 0.25 µg VSV‐G, 0.5 µg dR8.2, and 0.75 µg core plasmid were mixed with 4.5 µL PEI and added to the 293T cells for lentivirus production. The viral supernatant was harvested 36 and 72 h post‐transfection and passed through a 0.45 µm filtration system. Added viral particles to the target cell culture medium and incubated for 24 h. Replaced with fresh culture medium and cultured for 24 h before selection with 10 µg/mL blasticidin (#ant‐bl‐05, InvivoGene, USA) or 2 µg/mL puromycin (#P8833, Sigma, Germany) for knockdown cell screening. Knockdown and overexpression efficiencies were determined by qPCR or Western blotting.

### 4.12 Intracellular ROS Detection

The MDA‐MB‐231 cells and NK‐tdT cells were co‐cultured at a cancer‐to‐NK cell ratio of 2:1 for 0, 1, 2, and 3 h, respectively. The cells were washed with no phenol red DMEM (#21063029, Gibco, USA) to remove the NK‐tdT cells and stained MDA‐MB‐231 cells with 10 µm CM‐H2DCFDA (DCFDA; #C6827, Thermo Fisher Scientific, USA) for 30 min at 37°C. After staining, cells were washed and imaged using the Carl Zeiss microscope. The images were analyzed using ImageJ software.

### 4.13 MTT Assay

Cells (3,000 cells/well) were seeded into 96‐well plates. MTT reagent (#M2128, Sigma‐Aldrich, Germany) was added (10 µL/well) and incubated for 4 h at 37°C. After incubation, 100 µL of 10% SDS containing 0.01 m HCl was added, and the plate was incubated overnight. Absorbance was measured at 595 nm using a VICTOR X3 plate reader.

### 4.14 EdU Staining

NK cells (1.5 × 10^6^ cells/mL) were seeded in 60 mm culture dishes (1 mL/dish) and treated with 3 mL conditioned medium for 22 h. Incubating cells with 10 µm 5‐ethynyl‐2'‐deoxyuridine (BeyoClick EdU Cell Proliferation Kit with Alexa Fluor 488, #C0071L, Beyotime) for 2 h at room temperature. The cultured NK cells were centrifuged at 300 × g for 3 min to collect the cells. The cells were fixed in 4% paraformaldehyde for 15 min at room temperature. Permeabilized with 0.3% Triton X‐100 (#T8787, Sigma) in PBS at room temperature for 15 min or at 4°C overnight. Stained with Click‐iT reaction solution for 30 min and counterstained with 1× Hoechst 33342 (#C0071S‐6) for 10 min in the dark. The images were acquired using the Carl Zeiss microscope (20× objective) and analyzed using ImageJ software.

### 4.15 ELISA

231‐GFP and MCF7‐C3 cells (1 × 10^6^ cells/dish) were seeded in the 60 mm culture dish with 2.5 mL complete DMEM. Following cell attachment, NK‐tdT cells (5 × 10^5^ cells/dish) suspended in 2.5 mL of α‐MEM were added for co‐culture. Conditioned media were collected at predetermined time intervals (0, 3, 6, 9, 12, 18, 24, 36, and 48 h) and centrifuged at 300 × g for 10 min to remove debris. Human IFN‐γ in the supernatant was quantified using the ELISA MAX Deluxe Set (#430104, BioLegend, USA). Absorbance was measured at 450 nm (primary) and 570 nm (reference) on a Victor X3 plate reader (PerkinElmer). IFN‐γ concentrations (pg/mL) were determined based on a standard calibration curve.

### 4.16 Colony Formation Assay

Cancer cells (1000 cells/well) were seeded into 6‐well plates (#3516, Corning) with 2 mL complete medium and cultured for the indicated durations. Cells were washed twice with PBS, fixed with 4% paraformaldehyde (PFA, #158127, Sigma‐Aldrich, Germany) for 15 min at room temperature, and gently washed with PBS. Cells were stained with 0.5% crystal violet (#C6158, Sigma‐Aldrich, Germany) for 15 min. Excess stain was removed by three gentle PBS washes. Colonies were imaged using camera and quantified using ImageJ software.

### 4.17 Transwell Migration Assay

The experiment was performed using transwell inserts with 8 µm pores (#3422, Corning, USA). A total of 1×10^4^ cancer cells in 100 µL of serum‐free DMEM were seeded into the upper chamber, while the lower chamber was filled with 600 µL of complete DMEM supplemented with 10% FBS. Following 18 h incubation, non‐migrated cells were removed from the upper membrane surface with cotton swabs. Migrated cells on the lower membrane surface were fixed with 4% paraformaldehyde for 15 min, stained with 0.5% crystal violet for 15 min, and washed gently with PBS for three times. Membranes were excised and mounted on slides. Cells were imaged using a stereomicroscope (MVX10, Olympus). Migrated cells were quantified using ImageJ software.

### 4.18 Immunohistochemistry (IHC)

Tissue microarray slides of TNBC patients were obtained from Outdo Biotech Company (#TMA‐HBreD060Bc01, Shanghai, China). The experiments conducted on human tissue microarrays were authorized by the Ethics Committee of Shanghai Outdo Biotech Co., Ltd. (Reference Number: SHXC2021YF01). The experiments were performed using an IHC Detection Kit (#ab64264, Abcam, UK) following the manufacturer's protocol. Briefly, sections were deparaffinized and subjected to antigen retrieval (0.1 m sodium citrate buffer, pH 6.0). Endogenous peroxidase was blocked with 3% H_2_O_2_ and incubated with the primary antibody at 4°C overnight. Detected with HRP‐conjugated secondary antibody and visualized with DAB substrate. Counterstained with hematoxylin by Leica ST5020‐CV5030 Multistainer‐Coverslipper (Germany). Whole‐slide scanning was performed at 40× magnification on a NanoZoomer S60 scanner (#C13210‐01, HAMAMATSU, Japan). The IHC scores were calculated by adding the proportion score to the intensity score as previously described [48]. The Ki‐67 results were extracted by Outdo Biotech Company from the patient's pathology report. High expression was defined as IHC scores of 7–8 for IL8 and CXCL1, 6–8 for PD‐L1, 4–6 for CD56 and index of ≥50% for Ki‐67. All other scores were considered low expression.

### 4.19 Human Organoid Culture and Co‐Culture Assays

Two patient‐derived organoid (PDO) breast cancer lines used in this study were kindly provide by Prof. Chuxia Deng. These PDOs were established and characterized as previously reported [66]. Written informed consent was obtained from all patients prior to sample collection. The study protocol was approved by the ethics committees of the First Affiliated Hospital of Sun Yat‐sen University (ICE‐2017‐148) and Sun Yat‐sen University Cancer Center (308‐2015‐001), and Centro Hospitalar Conde de S. Januário (BSERE16‐APP010‐FHS).

For pharmacologic assays, PDOs were dissociated with trypsin, washed with PBS, and re‐embedded in Matrigel in 384‐well plates. After organoid re‐formation, cultures were treated with Atezolizumab (HY‐P9904, MCE), Reparixin (HY‐15252, MCE), or the combination for 4 days. Organoid viability was assessed using the CellTiter‐Lumi Plus Cell Viability Assay (C0068L, Beyotime) according to the manufacturer's protocol.

For NK‐PDO co‐culture assays, organoids were gently suspended in cold PBS to preserve structural integrity. NK cells were added at defined ratios based on the estimated number of tumor cells within organoids. The mixed cell suspension was resuspended in growth factor‐reduced Matrigel, seeded into culture plates, and allowed to polymerize. Bright‐field and fluorescence images were acquired daily to monitor organoid growth and NK cell dynamics.

After 72 h of co‐culture, organoids were washed with PBS, and viable cells were stained using Calcein AM (C2015L, Beyotime) according to the manufacturer's instructions. Images were captured using a Carl Zeiss Axio Observer Z1 inverted fluorescence microscope. The viability of cancer cells and NK cells was quantified using ImageJ software based on fluorescence area, calculated as follows:

$$\begin{array}{ccc} & & \mathrm{Live}\;\mathrm{cancer}\;\mathrm{cells}\;\%\\ & & \;=\frac{\mathrm{Total}\;\mathrm{live}\;\mathrm{cells}\;\mathrm{area}\;-\mathrm{live}\;\mathrm{NK}\;\mathrm{cells}\;\mathrm{area}}{\mathrm{Live}\;\mathrm{cancer}\;\mathrm{cells}\;\mathrm{area}\;\mathrm{in}\;\mathrm{monoculture}}\times 100 \end{array}$$

$$\mathrm{Live}\;\mathrm{NK}\;\mathrm{cells}\;\%=\frac{\mathrm{Live}\;\mathrm{NK}\;\mathrm{cells}\;\mathrm{area}}{\mathrm{Total}\;\mathrm{organoid}\;\mathrm{area}}\times 100$$

### 4.20 Statistical Analysis

All the results were obtained from three independent experiments or from five to six mice. All the data are presented as means ± standard deviation (SD). Statistical significance was determined using Student's *t‐*test, one‐way analysis of variance (ANOVA), or two‐way ANOVA. Significance was defined as follows: *p < 0.05, **p < 0.01, ***p < 0.001, and ****p < 0.0001. All analyses were performed using GraphPad Prism version 9.0.0 (GraphPad Software)

## Author Contributions

M.H.Y and K.Q.L. designed the study and the experiments. M.H.Y. conducted the experiments. H.M.Y., R.F.W. M.H. X.P.C., and C.X.D. helped with some of the experiments. M.H.Y, R.F.W., and K.Q.L. analyzed the data and wrote the manuscript.

## Conflicts of Interest

The authors declare no conflicts of interest.

## Supporting information




**Supporting File**: advs75655‐sup‐0001‐SuppMat.docx.

## Data Availability

The data that support the findings of this study are available on request from the corresponding author. The data are not publicly available due to privacy or ethical restrictions.
