# Supplementary material for: Triple‐Negative Breast Cancer Cells Utilize IL8 and CXCL1 to Suppress NK Cells’ Function and Facilitate Cancer Metastasis
Source: Adv Sci (Weinh). 2026 May 25;13(44):e75655. doi: 10.1002/advs.75655 (PMC13336074; doi:10.1002/advs.75655)
Supplement: Supplementary file 1 [file ADVS-13-e75655-s001.docx]

**Supporting Information**

**Triple-negative Breast Cancer Cells Utilize IL8 and CXCL1 to Suppress NK Cells’ Function and Facilitate Cancer Metastasis**

*Mingheng Yuan^1^, Hongmei Yang ^1^, Renfei Wu^1, 3^, Meng Hao^1, 4^, Xiangpeng Chu^1^, Chuxia Deng^1, 2^, Kathy Qian Luo^1, 2*^*

^1^Department of Biomedical Sciences, Faculty of Health Sciences, University of Macau, Taipa, Macao SAR 99078, China

^2^Ministry of Education Frontiers Science Center for Precision Oncology, University of Macau, Taipa, Macao SAR 99078, China

*****Corresponding author. Email: [kluo@um.edu.mo](mailto:kluo@um.edu.mo)

**Supplementary figures**

**Figure S1.** Patient-derived organoids from TNBC showed stronger resistance to NK cells’ cytotoxicity compared with non-TNBC. **A**) Cytotoxic effects of NK cells on breast cancer cell lines at different ratios for 6 h using calcein-AM assay. **B**) Cytotoxic effects of NK cells on breast cancer cell lines at a cancer-to-NK ratio of 1:1 for 6 h. **C, D**) Representative images of monoculture and co-culture of patient-derived organoid from TNBC (GZ2019062701T) and HER2-positive (BRC2018121702T) breast cancer patients with NK‑tdT cells at cancer‑ to‑NK ratios of 10:1 or 10:2 for 72 h. Scale bar, 100 μm. The results represent the means ± SD from three independent experiments. Significant differences were determined by Student’s t-test or two-way ANOVA. **p* < 0.05, ***p* < 0.01, ****p* < 0.001.

**Figure S2.** Conditioned medium (CM) of TNBC can inhibit the proliferation of NK cells. **A**) 3D culture of NK-tdT cells in the CM from various breast cancer cell lines. Scale bar, 200 μm. **B**) Quantification of NK-tdT cells’ viability cultured in CM from breast cancer compared with DMEM. **C**) Representative images of EdU-positive cells following treatment with DMEM or CM from MCF7-C3, 231-GFP, and BT549-clover. Scale bar, 50 μm. **D**) The proportion of EdU-positive NK cells after CM treatment. **E**) Concentration of IFN-γ in the co-cultured CM from MCF7-C3 and 231-GFP cells interacting with NK-tdT cells was measured. **F**) Fluorescence intensity of NK-tdT cells after co-culture with breast cancer cell lines for 48 h. The results represent the means ± SD from three independent experiments. Significant differences were determined by Student’s t-test, one-way ANOVA, or two-way ANOVA. **p* < 0.05, ***p* < 0.01, *****p* < 0.0001, ns, not significant.

**Figure S3.** Top 10 cytokines expression validation in breast cancer cell lines. **A**) Relative mRNA levels of potential target genes in breast cancer cell lines measured by qPCR. **B**) The normalized transcript per million (nTPM) of potential target genes in breast cancer cell lines. These data are from “The Human Protein Atlas” database.

**Figure S4.** The correlation among IL8, CXCL1, and PD-L1 expressions and their relationship with NK cell infiltration**. A**) Heatmap showing the FPKM values of seven cytokines that can bind to CXCR1/2. **B**) Expression levels of CXCL3 in human normal and breast cancer tissue (BRCA) based on breast cancer subclasses from TCGA samples, n=833 samples (UALCAN webtool). **C**) The correlation between any two of IL8, CXCL1, and PD-L1 expression levels in TNBC performed by the TIMER 2.0 platform. n=191 human TNBC samples. **D**) The correlation between IL8, CXCL1, and PD-L1 expression levels with NK cell infiltration in TNBC performed by the TIMER 2.0 platform. n=191 human TNBC samples. **E**) The correlation between IL8, CXCL1 and PD-L1 expression levels with NK cell infiltration in breast cancer was performed by the TIMER 3.0 platform. n=1086 human breast cancer samples. Significant differences were determined by Student’s t-test or Spearman correlation analysis. *****p* < 0.0001.

**Figure S5.** Knockdown of IL8 and CXCL1 reduces PD-L1 expression and increases NK cell cytotoxicity in BT549 cells**. A-C**) qPCR showing that knockdown of any single gene among IL8, CXCL1, and PD-L1 leads to a reduction in the mRNA levels of the other two genes in BT549-clover cells. **D, E**) Assessment of NK cell cytotoxic efficiency against BT549-clover knockdown cells in a 3D co-culture system at a cancer-to-NK cell ratio of 2:1 for 48 h. Scale bar, 200 μm. **F, G**) Assessment of NK cell cytotoxic efficiency against BT549-clover knockdown cells in a 2D co-culture system at a cancer-to-NK cell ratio of 2:1 for 48 h. Each image was generated by stitching 36 individual images. Scale bar, 1 mm. **H, I**) 3D culture showing the effect of CM from BT549-clover knockdown cells on NK cell activity. Scale bar, 200 μm. **J**) Schematics illustrating the anatomical location of primary and metastatic tumors. **K, L**) Representative images of iliac lymph node metastasis and the quantified percentage of mice with lymphatic node metastasis. Scale bar, 1 mm. **M, N**) Representative images of lung metastasis and quantified results of GFP+ colonies per left lung. Scale bar, 2 mm. **O**) Tumor weight of breast primary tumors was measured on day 42. **P**) Quantification of body weight for each mouse. The results represent the means ± SD from three independent experiments. Significant differences were determined by one-way ANOVA. **p* < 0.05, ***p* < 0.01, ****p* < 0.001, *****p* < 0.0001, ns, not significant.

**Figure S6.** Overexpression of IL8 and CXCL1 desensitized MCF7-C3 and HCC1937-clover cells to NK killing and enhanced NK inhibition. **A**) Western blot results indicate the protein levels of IL8 and CXCL1 in OE-IL8 and OE-CXCL1 cells derived from MCF7-C3 cells. **B**) Overexpression efficiency of IL8 and CXCL1 in MCF7-C3 cells were validated by qPCR. **C**) Representative fluorescence images from 3D co-culture (left), 2D co-culture (center), and NK-tdT cells treated with conditioned medium (CM to NK-tdT, right). In co-culture groups, MCF7-C3 overexpression cells were co-cultured with NK-tdT cells at a 5:1 ratio for 48 h. NK-tdT cells incubated with conditioned medium derived from MCF7-C3 overexpression cells. Scale bars: 200 µm (3D, CM), 100 µm (2D). **D**) Quantified results corresponding to the 3D co-culture experiment shown in Figure S6C. **E**) Quantified results corresponding to the 2D co-culture experiment shown in Figure S6C. **F**) Quantified results corresponding to the CM treatment experiment shown in Figure S6C. **G**) Overexpression efficiency of IL8, CXCL1, and PD-L1 in HCC1937-clover cells was validated by qPCR. **H, I**) Fluorescence images and quantified results of 3D co-culture showing HCC1937-clover overexpressing cells co-cultured with NK-tdT cells at a cancer-to-NK cell ratio of 2:1 for 48 h. Scale bar, 200 μm. **J, K**) Fluorescence images and quantified results of NK-tdT cells after 48 h incubation with CM derived from 231-GFP overexpression cells. Scale bar, 200 μm. The results represent the means ± SD from three independent experiments. Significant differences were determined by one-way ANOVA. **p* < 0.05, ***p* < 0.01, ****p* < 0.001, *****p* < 0.0001, ns, not significant.

**Figure S7.** 231-MBC cells exhibited stronger colony formation and migration ability compared to 231-GFP cells**. A**) MTT assay to compare the cell proliferation ability between 231-MBC and 231-GFP cells. **B**, **C**) 3D sphere formation assay to compare the cell proliferation ability between 231-MBC and 231-GFP cells. Scale bar, 200 μm. **D**, **E**) Representative images and corresponding quantitative data illustrating the colony formation ability of 231-GFP and 231-MBC cells. Scale bar, 5 mm. **F, G**) Representative images and quantitative results of the migration capacity between 231-GFP and 231-MBC cells. Scale bar, 200 μm. The results represent the means ± SD from three independent experiments. Significant differences were determined by Student’s t-test or two-way ANOVA. **p* < 0.05, ****p* < 0.001, ns, not significant.

**Figure S8.** 231-MBC cells rely on the IL8/CXCL1-CXCR1/2 signaling pathway to acquire enhanced metastatic capacity**. A**) qPCR results of the knockdown efficiency of IL8, CXCL1, PD-L1, CXCR1, and CXCR2 in the 231-MBC cells transfected with shRNA. **B, C**) Representative images and quantitative data of the colony formation assay from 231-MBC cells after knocking down the IL8, CXCL1, and CXCR1/2 expression. **D, E**) Representative images and quantitative results of the migration assay from 231-MBC cells after knocking down the IL8, CXCL1, and CXCR1/2 expression. **F, G**) Representative images and statistical results of 231-MBC cells co-cultured with NK-tdT cells at a ratio of 1:1 for 48 h under 2D conditions following IL8, CXCL1, and CXCR1/2 knockdown. Each image was generated by stitching 36 individual images. Scale bar, 1 mm. **H, I**) Representative images and statistical analysis of NK-tdT cells cultured in conditioned medium derived from IL8, CXCL1, and CXCR1/2 knockdown 231-MBC cells for 48 h. Scale bar, 200 μm. The results represent the means ± SD from three independent experiments. Significant differences were determined by Student’s t-test or one-way ANOVA. **p* < 0.05, ***p* < 0.01, ****p* < 0.001, *****p* < 0.0001.

**Figure S9.** CXCL1 and IL8 represent the tumor-associated CXCR1/2 ligands in TNBC**.** **A**) qPCR showing that knockdown of any single gene among IL8, CXCL1, and CXCR1/2 leads to a reduction in the mRNA levels of CXCL1, CXCL2, and IL8 in 231-MBC cells. **B**) qPCR showing that knockdown of IL8 or CXCL1 leads to a reduction in the mRNA levels of CXCL1, CXCL2, CXCL3, and IL8 in BT549-clover cells. The results represent the means ± SD from three independent experiments. Significant differences were determined by one-way ANOVA. **p* < 0.05, ***p* < 0.01, ****p* < 0.001, *****p* < 0.0001, ns, not significant.

**Figure S10.** Co-culturing with NK cells upregulates the expression of IL8, CXCL1, and PD-L1 via the NF-κB, JAK−STAT1, and ERK/JNK−AP-1 signaling pathways in 231-GFP cells and BT549 cells**. A**) Western blot results showing the total protein levels of IκBα, NF-κB, JAK1, JAK2, TYK2, STAT1, ERK1/2, JNK, c-FOS, and c-JUN in 231-GFP cells before and after co-culture with NK cells. **B**) Western blot results revealing the total protein levels of relevant signaling molecules in 231-GFP cells mono- or co-cultured with NK-tdT cells at a cancer-to-NK cell ratio of 2:1 for 6 h, with or without ROS inhibitor PG (20 μM) and NAC (5 mM). **C**) Western blot results of total protein levels of relevant signaling molecules in 231-GFP cells mono- or co-cultured with NK-tdT cells at a cancer-to-NK cell ratio of 2:1 for 6 h, with or without JAK inhibitor Peficitinib (10 μM). **D**) Western blot results of STAT1, p-STAT1, IL8, CXCL1, and PD-L1 expression in cells treated with or without STAT1 inhibitor Fludarabine (5 μM) for 6 h under mono- or co-culture conditions with NK cells. **E**) Western blot results showing the protein levels of relevant signaling molecules in BT549-clover cells before and after co-culture with NK cells at a cancer-to-NK cell ratio of 2:1 for 6 h.

**Figure S11.** IL8 and CXCL1 were associated with increased tumor metastasis, proliferation, and decreased NK cells infiltration. **A, B**) Knockdown of CXCR1/2, PD-L1, or both attenuated lung metastasis of 231-MBC cells in nude mice. n=6, Scale bar, 2 mm. **C**) High expressions of IL8/CXCL1 correlated with a low number of CD56⁺ NK cells’ infiltration in TNBC tumors with both high and low levels of PD-L1. **D**) Correlation between the expression of IL8, CXCL1, and PD-L1 with Ki-67 in tumors of TNBC patients. Significant differences were determined by Student’s t-test or one-way ANOVA. **p* < 0.05, ***p* < 0.01, ns, not significant.

**Supplementary tables**

**Table S1. List of primers for qPCR.**

| **Gene name** | **Direction** | **Primer sequence (5’-3’)** |
| --- | --- | --- |
| IL8 | Forward  Reverse | ACTGAGAGTGATTGAGAGTGGAC  AACCCTCTGCACCCAGTTTTC |
| CXCL1 | Forward  Reverse | ACTCTACCTGCACACTGTCCTA  CATTTGCTTGGATCCGCCAG |
| PD-L1 | Forward  Reverse | GGACAAGCAGTGACCATCAAG  CCCAGAATTACCAAGTGAGTCCT |
| GAPDH | Forward  Reverse | CCCCACCACACTGAATCTCC  GTACATGACAAGGTGCGGCT |
| CSF3 | Forward  Reverse | GCTGCTTGAGCCAACTCCATA  GAACGCGGTACGACACCTC |
| CSF2 | Forward  Reverse | TCCTGAACCTGAGTAGAGACAC  TGCTGCTTGTAGTGGCTGG |
| CD40 | Forward  Reverse | TTGGGGTCAAGCAGATTGCTA  GCAGATGACACATTGGAGAAGA |
| TGFβ2 | Forward  Reverse | CCATCCCGCCCACTTTCTAC  AGCTCAATCCGTTGTTCAGGC |
| CXCL2 | Forward  Reverse | TGAATCTACTTGCACACTCTCCC  TACATTTCCCTGCCGTCACAT |
| CXCL3 | Forward  Reverse | TTCTAGGGACAGCTGGAAAGG  TCCCCACCCTGTCATTTATCA |
| CXCL5 | Forward  Reverse | AGCTGCGTTGCGTTTGTTTAC  TGGCGAACACTTGCAGATTAC |
| CXCL6 | Forward  Reverse | AGAGCTGCGTTGCACTTGTT  GCAGTTTACCAATCGTTTTGGGG |
| CXCL7 | Forward  Reverse | TGCTCTGGCTTCCTCCACCAAA  ACACATGCAGCGGAGTTCAGCA |
| IL1β | Forward  Reverse | ATGATGGCTTATTACAGTGGCAA  GTCGGAGATTCGTAGCTGGA |
| IL11 | Forward  Reverse | CGGACAGGGAAGGGTTAAAG  CAGGCGGCAAACACAGTTC |
| CXCR1 | Forward  Reverse | TCCTTTTCCGCCAGGCTTACCA  GGCACGATGAAGCCAAAGGTGT |
| CXCR2 | Forward  Reverse | CCTGTCTTACTTTTCCGAAGGAC  TTGCTGTATTGTTGCCCATGT |

**Table S2. List of antibodies for Western blot analysis.**

| **Antibody name** | **Company** | **Catalog #** | **Application** |
| --- | --- | --- | --- |
| Erα, Rabbit | CST | 8644 | WB (1:1000) |
| HER2/ErbB2, Mouse | CST | 2248 | WB (1:1000) |
| IL8, Rabbit | CST | 94407 | WB (1:500) |
| IL8, Mouse | Abcam | Ab282027 | IHC (1:50) |
| CXCL1, Rabbit | ThermoFisher | PA5-86508 | WB (1:500); IHC (1:100) |
| PD-L1, Rabbit | CST | 13684 | WB (1:1000) |
| GAPDH, Rabbit | CST | 2118 | WB (1:1000) |
| CXCR1, Mouse | Abcam | Ab89251 | WB (1:1000) |
| CXCR2, Mouse | Abcam | Ab89254 | WB (1:1000) |
| IκBα, Mouse | CST | 4814 | WB (1:1000) |
| p- IκBα, Mouse | CST | 9246 | WB (1:1000) |
| NF-κB, Rabbit | CST | 8242 | WB (1:1000) |
| p-NF-κB, Rabbit | CST | 3033 | WB (1:1000) |
| JAK1, Rabbit | CST | 3344 | WB (1:1000) |
| p-JAK1, Rabbit | CST | 74129 | WB (1:1000) |
| JAK2, Rabbit | CST | 3230 | WB (1:1000) |
| p-JAK2, Rabbit | CST | 8082 | WB (1:1000) |
| TYK2, Rabbit | CST | 14193 | WB (1:1000) |
| p-TYK2, Rabbit | CST | 68790 | WB (1:1000) |
| STAT1, Rabbit | CST | 14994 | WB (1:1000) |
| p-STAT1, Rabbit | CST | 7649 | WB (1:1000) |
| ERK1/2, Rabbit | CST | 4695 | WB (1:1000) |
| p-ERK1/2, Rabbit | CST | 4370 | WB (1:1000) |
| JNK, Rabbit | CST | 9252 | WB (1:1000) |
| p-JNK, Rabbit | CST | 9251 | WB (1:1000) |
| c-FOS, Rabbit | CST | 31254 | WB (1:1000) |
| p-c-FOS, Rabbit | CST | 5348 | WB (1:1000) |
| c-JUN, Rabbit | CST | 9165 | WB (1:1000) |
| p-c-JUN, Rabbit | CST | 3270 | WB (1:1000) |
| AKT, Rabbit | CST | 4691 | WB (1:1000) |
| p-AKT (Ser473), Rabbit | CST | 4060 | WB (1:1000) |
| BCL-2, Mouse | CST | 15071 | WB (1:1000) |
| STAT3, Mouse | CST | 9139 | WB (1:1000) |
| p-STAT3, Rabbit | CST | 9134 | WB (1:1000) |
| NKG2D, Rabbit | ThermoFisher | PA5-85205 | WB (1:1000) |
| NKG2A, Rabbit | Abcam | Ab260035 | WB (1:1000) |
| TRAIL, Rabbit | CST | 3219 | WB (1:1000) |
| IFN-γ, Rabbit | LSBio | LS-C809500 | WB (1:1000) |
| Perforin, Mouse | Biolegend | 353316 | WB (1:1000) |
| NCAM1 (CD56) , Rabbit | CST | 99746 | IHC (1:100) |
| Goat anti-Rabbit IgG (H+L) 2^nd^ Ab, HRP | Bio-Rad | 1706515 | WB (1:5000) |
| Goat anti-Mouse IgG (H+L)- 2^nd^ Ab, HRP | Invitrogen | 31430 | WB (1:5000) |

*CST, Cell Signaling Technology, USA.

**Table S3. List of shRNA**

| **shRNA name** | **Target sequence (5’-3’)** |
| --- | --- |
| shIL8 #1 | CCAGATGCAATACAAGATTCC |
| shIL8#2 | TGCGCCAACACAGAAATTATT |
| shCXCL1#1 | ATATTTTAGGTGTAAAATAAT |
| shCXCL1#2 | ATAGTTAAGAAAATCATCGAA |
| shPD-L1 | AACACAACAACTAATGAGATT |
| shCXCR1 | GATGTCTACCTGCTGAACCTG |
| shCXCR2 | TCAAAATTCATATGTCTCAGCA |

**Table S4. List of overexpression vectors**

| **Vector name** | **Transcript ID** | **Sequence length** |
| --- | --- | --- |
| IL8 | NM_000584.4 | 300 bp |
| CXCL1 | NM_001511.4 | 324 bp |
| PD-L1 | NM_014143.4 | 873 bp |
